# Flexible Bifunctional Electrode for Alkaline Water Splitting with Long-Term Stability

**DOI:** 10.1021/acsami.3c12944

**Published:** 2024-03-01

**Authors:** Abhijit Ganguly, Ruairi J. McGlynn, Adam Boies, Paul Maguire, Davide Mariotti, Supriya Chakrabarti

**Affiliations:** † School of Engineering, Ulster University, Belfast BT15 1AP, Northern Ireland, U.K.; ‡ Department of Engineering, University of Cambridge, Cambridge CB2 1PZ, U.K.

**Keywords:** bifunctional and flexible electrode, macroscopically assembled carbon nanotube (CNT) ribbons, nickel oxides (NiO) quantum dots (QDs), plasma-induced nonequilibrium electrochemistry (PiNE), hydrogen evolution reaction (HER), oxygen evolution reaction (OER), water electrolysis, overall water splitting (OWS) in alkaline media, alkaline electrolyzer cell, long-term OWS stability

## Abstract

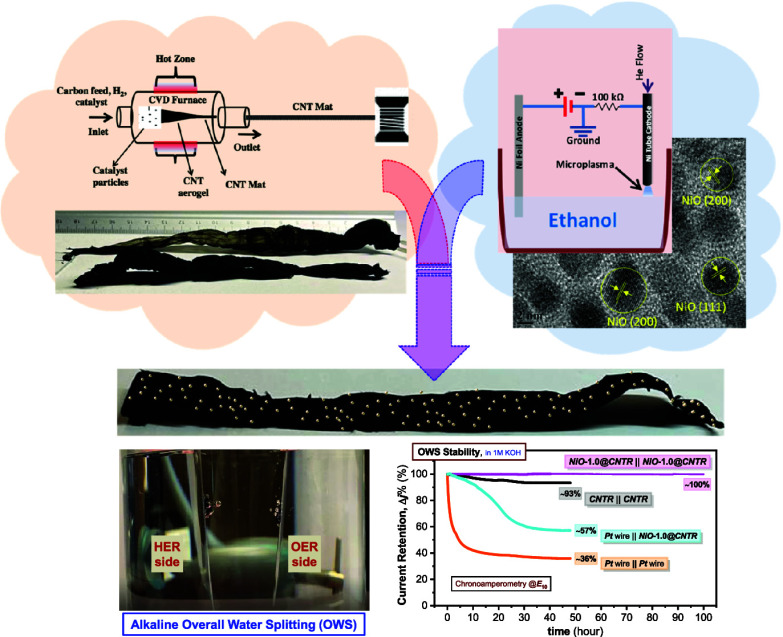

Progress in electrochemical water-splitting devices as future renewable and clean energy systems requires the development of electrodes composed of efficient and earth-abundant bifunctional electrocatalysts. This study reveals a novel flexible and bifunctional electrode (***NiO@CNTR***) by hybridizing macroscopically assembled carbon nanotube ribbons (***CNTRs***) and atmospheric plasma-synthesized NiO quantum dots (QDs) with varied loadings to demonstrate bifunctional electrocatalytic activity for stable and efficient overall water-splitting (OWS) applications. Comparative studies on the effect of different electrolytes, e.g., acid and alkaline, reveal a strong preference for alkaline electrolytes for the developed ***NiO@CNTR*** electrode, suggesting its bifunctionality for both HER and OER activities. Our proposed ***NiO@CNTR*** electrode demonstrates significantly enhanced overall catalytic performance in a two-electrode alkaline electrolyzer cell configuration by assembling the same electrode materials as both the anode and the cathode, with a remarkable long-standing stability retaining ∼100% of the initial current after a 100 h long OWS run, which is attributed to the “synergistic coupling” between NiO QD catalysts and the CNTR matrix. Interestingly, the developed electrode exhibits a cell potential (*E*_10_) of only 1.81 V with significantly low NiO QD loading (83 μg/cm^2^) compared to other catalyst loading values reported in the literature. This study demonstrates a potential class of carbon-based electrodes with single-metal-based bifunctional catalysts that opens up a cost-effective and large-scale pathway for further development of catalysts and their loading engineering suitable for alkaline-based OWS applications and green hydrogen generation.

## 1 Introduction

Alternative clean energy sources are essential to avoid the harmful impacts of fossil fuel^1,2^ to produce a cleaner environment. In this regard, electrochemical water electrolysis is a promising sustainable technology for the large-scale production of hydrogen as a clean fuel.^3−6^ Industrial-scale application of water electrolyzer cells is difficult without replacing the expensive platinum-based electrode for hydrogen evolution reaction (HER) and electrodes based on Ir/Ru for oxygen evolution reaction (OER).^7^ It is necessary to develop alternative earth-abundant, low-cost, stable, and efficient electrodes made of non-noble metal compounds.^4−8^ Likewise, the development of a bifunctional electrocatalyst is becoming increasingly important to achieve a significant leap in this technology to avoid the complexity of using different electrodes for HER and OER in the electrolyzer cell.^4−6,8−16^ Generally different electrodes for HER and OER show electrolyte preferences for their best performance, e.g., Ir/Ru-based OER catalysts prefer an alkaline solution, while Pt-based HER catalysts exhibit high activities in acidic electrolytes.^6−8,14,17^ In addition to the high cost, Pt-based electrodes deteriorate due to their time-dependent drift^18^ and CO deactivation,^19^ which also justifies the development of an alternative bifunctional electrocatalyst with long-term operation stability throughout the lifetime of the single-electrolyte-based water electrolyzer cell.

In this work, we report the development of ***NiO@CNTR*** hybrids with varied loadings of NiO QDs on the surface of CNTR as a potential class of a carbon-based bifunctional electrode with superior electrocatalytic activity and long-standing operation stability for overall water splitting (OWS) in alkaline electrolyzer cells. We have synthesized macroscopic assemblies of carbon nanotubes (CNTs) in the form of a ribbon (***CNTR***) directly spun from the chemical vapor deposition (CVD) reactor,^20−23^ which provides a competitive advantage over the conventional technique where CNTs are embedded in a host matrix and suffer from their poor dispersibility.^24,25^ Stable and quantum-confined NiO QDs in the form of colloids were synthesized by atmospheric plasma-induced nonequilibrium electrochemistry (PiNE).^26^ The NiO QD colloids were then spray-coated onto the CNTR surface to make ***NiO@CNTR*** electrodes. The proposed bifunctional electrode showed an excellent OWS catalytic performance (cell potential window, *E*_10_, of 1.81 V), significantly better than a metallic ***Pt*** wire electrode (*E*_10_ of 2.15 V). The chronoamperometric study revealed that the ***NiO@CNTR*** electrode has long-term current stability, close to 100% over 100 h of OWS run, much higher than the ***Pt*** wire electrode, which showed only a 36% current retention over 48 h.

We also demonstrated that the pristine electrode (***CNTR***, zero loading of NiO QDs) exhibited promising bifunctional electrocatalytic performances for alkaline-based OWS with an *E*_10_ of 2.11 V, better than that of the ***Pt*** wire electrode (∼2.15 V) and with significantly better stability (retaining ∼94% of the initial current after a 48 h long run) than ***Pt*** (retaining only 36%), suggesting its potential application for a practical two-electrode OWS device in alkaline electrolyte. A comparative study of HER activity in acidic and alkaline media revealed that the ***CNTR*** and ***NiO@CNTR*** electrodes prefer alkaline media over acid.

The novelty of this work primarily lies with the proposed electrode materials composed of CNTR and PiNE-synthesized highly stable NiO QDs. The electrode support material CNTR is novel, which is the bulk form of network structured carbon nanotubes and holds superior properties such as chemical robustness, high electrical conductivity, and compatibility with various metal and metal oxide nanostructured catalysts. The synthesis process of CNTR is scalable with no length limitation, and its macroscopic form provides the flexibility to engineer these materials in terms of shape and size. Furthermore, the binder-free direct attachment of NiO QDs with CNTR provides a clear interface between the catalyst and the electrode support and shows excellent chemical and mechanical stability of ***NiO@CNTR*** hybrids, which is beneficial for achieving the observed “long-term” stability in OWS performance. Another novelty of this work is the successful application of the spray-coating technique for the desirable catalyst loading onto CNTR, which gives the advantage of the ease, rapidness, and scalability of the electrode fabrication. This study demonstrates the proposed ***NiO@CNTR*** electrode as a potential class of novel carbon-based electrodes and CNTR as an effective support for further catalyst development suitable for alkaline-based OWS applications and green hydrogen generation.

## 2 Results and Discussion

### 2.1 Macroscopically Assembled CNT Ribbon, CNTR

**Figure 1:**
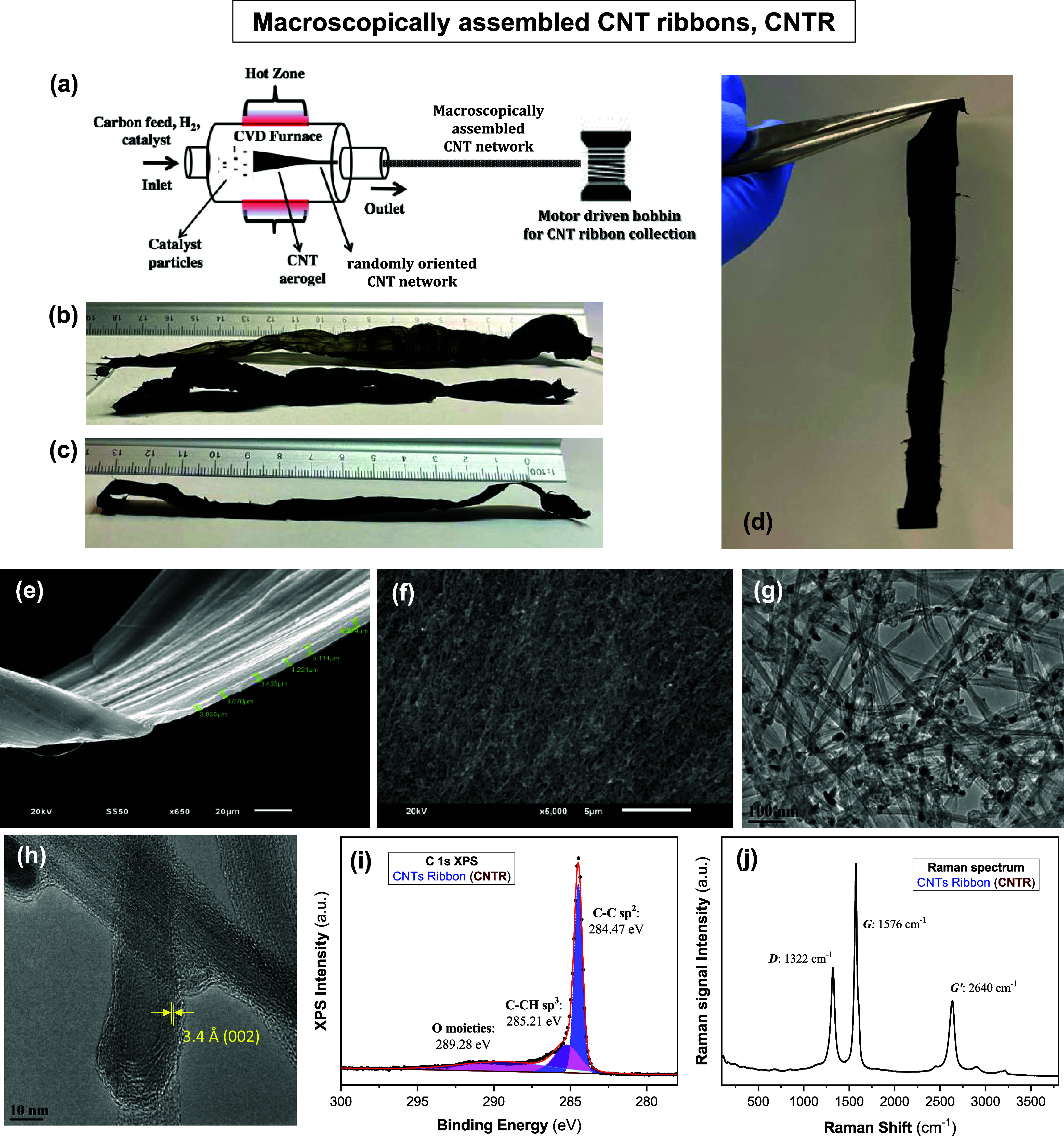
Macroscopically assembled CNT ribbon, CNTR. (a) Schematic diagram of the CVD system used for the winding of continuous CNT ribbons (CNTR). Representative images of (b) typical as-synthesized 3D ensemble of the randomly oriented CNT network and (c-d) flat and flexible CNTR after compression. (e) SEM image of a CNTR showing thickness in the range of 3–4 μm. (f) SEM image of the top surface of a CNTR at higher magnification showing dense packing and random orientation of the nanotubes. (g) TEM image and (h) high-resolution TEM image of the CNTR with an interlayer spacing of 3.4 Å, corresponding to the (002) plane. (i) High-resolution XPS spectrum of C 1s and (j) Raman spectrum of pristine CNTR.

Macroscopically assembled CNT ribbons (CNTRs) were synthesized using a floating catalyst-assisted thermal CVD technique.^20,22,23^ This method can produce macroscopic assemblies of individual CNTs in a single step, where the CNTR can be directly drawn from the CVD furnace chamber without an obvious limit to the length of the material. Figure 1a shows the schematic diagram of the CVD system used for the winding of continuous CNTR from the chamber. In the “Section 4”, a detailed principle and procedure of the CNTR synthesis is illustrated, following our earlier publication by Brunet et al.^20^

In the Supporting Information (SI, Section S2), Supporting Figure S1a,b demonstrates the direct dragging of the ribbon-like macroscopically assembled CNT network from the furnace chamber. Figure 1b represents a typical as-synthesized 3D ensemble of the randomly oriented CNT network (Figure S1c,d), which was further processed to form a flat and compact fabric-like material (shown in Figure 1c,1d) prior to any characterization or application. Figure S1e–k reveals the flexible and lightweight nature of a typical CNTR (also see the Supporting Video SV1_CNTR). The detailed compaction process is mentioned in the Section 4 under “Section 4.2”. Depending on the pressure applied, the thickness of the final CNTR material can be controlled. In this study, the CNTR thickness was maintained within 3–5 μm. The material (CNTR) and its synthesis process were extensively characterized in our previous publication.^27^

Figure 1e represents a scanning electron microscopy (SEM) image of a flat and compact CNTR sample. The representative thickness of the CNTR used in this report is between 3 and 5 μm. The top-view SEM image of a CNTR (Figure 1f) shows dense packing and randomly entangled individual CNTs forming a ribbon composed of a three-dimensional (3D) macroscopic assembly. A representative transmission electron microscopy (TEM) image, shown in Figure 1g, depicts individual nanotubes with an average diameter of 20 nm. Typical high-resolution TEM (HRTEM) observation (Figure 1h) indicates that the CNTs are multiwalled nanotubes with an interlayer spacing of 3.4 Å, corresponding to the (002) plane. Further, HRTEM investigation (Figure S2a,b) and the electron energy loss spectroscopy (EELS) spectrum mapping images (Figure S2c) have also revealed the presence of Fe catalysts encapsulated inside the nanotubes.

X-ray photoelectron spectroscopic (XPS) analysis of the pristine CNTR was performed to understand the chemical composition of the CNTs. Deconvolution of the high-resolution XPS spectrum of the C 1s spectrum, shown in Figure 1i, resolved three distinct peaks centered at 284.47, 285.21, and 289.28 eV. The peak at 284.47 eV can be attributed to the aromatic carbon bonding (C–C sp^2^)^28^ from the hexagonal walls of the CNTs.^28^ The peak at 285.2 eV refers to the C–CH sp^3^ bond, which is related to defects in the aromatic structure.^29^ The broad peak centered at 289.28 eV appears due to the combined effect of different surface chemical bindings of HO–C=O or COOH, C–O, C=O, and O–C=O.^28^

Raman spectroscopy has proven to be an effective and noninvasive tool to obtain experimental information about the electronic and vibrational properties of the CNTs.^30,31^Figure 1j shows a typical Raman spectrum of pristine CNTR, which depicts three peaks at 1322, 1576, and 2640 cm^–1^, corresponding to the D-band, the G-band, and the G′-band, respectively. Since the presence of defects in the CNT lattice enables elastic scattering processes, the area or intensity of the D-band scales with the concentration of defects within a CNT sample and can be used as an indicator of poor crystallinity.^32^ The G-band peak area or intensity is a measure of carbon nanotube graphitization and is related to the graphite tangential *E*_2*g*_ Raman active mode where the two carbon atoms in the graphene unit cell are vibrating tangentially one against the other. The G-band to D-band ratio indicates the graphitization to defect content ratio of the synthesized CNTs. Here, the G-band to D-band ratio (ratio of two peak areas) is found to be 2.29 ± 0.03, representing a high degree of graphitization in the as-synthesized CNTR. This high degree of graphitization suggests high purity^23,30^ with large sp^2^ domains^28^ on the hexagonal walls of the nanotubes of the CNTR. This provides structural stability and extended electrocatalytic active sites,^33,34^ essential for high-performance catalytic electrodes.

### 2.2 NiO Quantum Dots (QDs) and ***NiO@CNTR*** Electrodes

NiO quantum dots (QDs) were synthesized using atmospheric plasma-induced nonequilibrium electrochemistry (PiNE).^26,35−37^ The digital image and the schematic diagram of the PiNE setup are shown in Figure S4a,b, respectively. Full details of the NiO QD synthesis procedure are illustrated in the Section 4 (under “Section 4.3”). As demonstrated in our earlier publication by Chakrabarti et al.,^26^ the PiNE-synthesized NiO QDs possess a narrow size distribution with an average diameter of 3 nm in the quantum confinement regime (Figure S4c). The lattice spacing measurements and the SAED pattern obtained from HRTEM images (Figure S4d,e) show clear lattice fringes corresponding to the (200) and (111) planes of the cubic NiO phase [JCPDS-No.: 47-1049]. Elemental characterization via XPS analysis clearly reveals the presence of the Ni 2p^3/2^ peak (Figure S4f). After deconvolution, three peaks can be extracted from the spectrum, which is in good agreement with previously reported NiO films synthesized by various techniques.^26,38−40^ The XPS peak centered at 853.5 eV indicates the presence of Ni^2+^ in the Ni–O octahedral bonding of cubic rocksalt NiO.^39,40^ The peak centered at 855.1 eV can be attributed to the vacancy-induced Ni^3+^ ion^41^ or nickel hydroxides and oxyhydroxides.^42^ Finally, the broad peak centered at 860.1 eV is due to the shakeup process^39^ in the NiO structure.

For electrode preparation, the CNTR pieces were manually interlaced with adhesive copper tape and nickel conducting paste, enlarging the electrical contact for current collection. Subsequently, the whole assembly was laminated, maintaining a window (for the electrochemical reaction) of a 0.45 cm diameter, as shown in Figure S3a. Prior to the electrocatalytic measurements, the laminated CNTR electrodes were tested and optimized by employing conventional electrochemical characterizations (details are given in SI, Section S2 and Figure S3b–d). No leakage through lamination and wetting of the CNTR surface were observed, which confirms zero interference of the metal contact (adhesive Cu tape or Ni conducting paste) with the electrolyte solution.

For fabrication of NiO QD-coated CNTR electrodes (***NiO@CNTR***), the PiNE-synthesized NiO QD-ethanol colloids were spray-coated onto the laminated CNTR electrodes at three different volume concentrations, 66 μg/mL (as-synthesized colloids, symbolized as *NiO*-1.0), 33 μg/mL (two times diluted, named as *NiO*-0.5), and 6.6 μg/mL (diluted by ten times, symbolized as *NiO*-0.1). Here, it is crucial to confirm that, for the symbol used for the NiO QD colloids, the numeric value represents the dilution factor, not any chemical composition. Detailed spray-coating parameters and NiO QD loading concentration are given in the “Section 4” and Table S1 of the Supporting Information. In summary, the three different ***NiO@CNTR*** electrodes, namely ***NiO*****-1.0*****@CNTR***, ***NiO*****-0.5*****@CNTR***, and ***NiO*****-0.1*****@CNTR***, were loaded by 83, 42, and 8.3 μg/cm^2^ of NiO QDs, respectively. For comparison, the pristine CNT ribbon was also tested as an electrode (***CNTR***), representing its electrocatalytic activity without any additional catalyst. Notably, in this study, the maximum loading of NiO QDs as a catalyst (∼83 μg/cm^2^ for the ***NiO*****-1.0*****@CNTR*** electrode) is 3–20 times lower than the average catalyst loading values reported in the literature.^9,10,13,14,16,43−47^ The NiO QD loading was optimized to achieve the minimum possible concentration for maximum performance, which will facilitate its applicability on an industrial scale, favoring the low-weight and low-cost approach.

**Figure 2:**
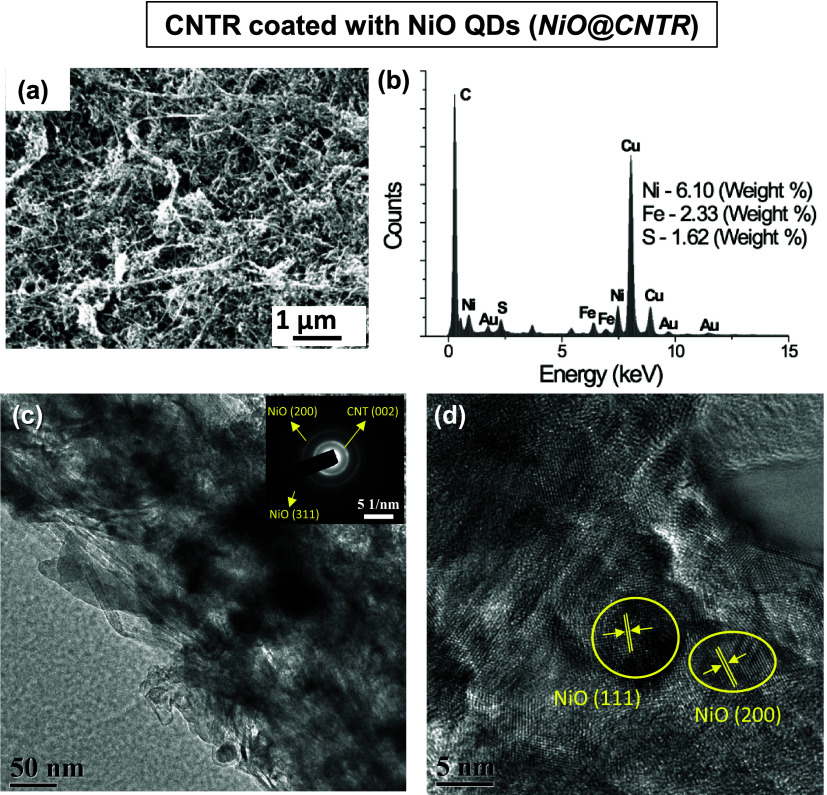
Nickel oxide (NiO) quantum dot (QD)-coated CNTR. (a) SEM image of a representative CNTR coated with NiO QDscolloids (***NiO*****-1.0**). (b) EDX spectrum confirming the presence of NiO QDs on CNTR. Fe and S signals originate from the residual catalysts, and Cu originates from the TEM grid. (c) TEM micrograph of ***NiO@CNTR***, and the inset shows the crystalline phase of the cubic NiO and (002) plane of CNTs. (d) Representative lattice-resolved HRTEM image of ***NiO@CNTR*** showing the high-resolution view of NiO particles on the CNTR matrix.

The SEM image in Figure 2a shows NiO QDs coated on a CNTR, and the TEM-based EDS spectrum (Figure 2b) of ***NiO@CNTR*** reveals the presence of a Ni element in addition to carbon, confirming the presence of NiO QDs on the surface of CNTR. The TEM micrograph (Figure 2c) and the corresponding SAED pattern (inset, Figure 2c) of a ***NiO@CNTR*** sample indicate both the crystalline phase of cubic NiO and the (002) plane of CNT. The lattice-resolved HRTEM image shown in Figure 2d displays lattice fringes corresponding to the (200) and (111) planes of the cubic NiO phase [JCPDS-No.: 47-1049], clearly indicating that the NiO QDs were successfully integrated on the CNTR. For the ***NiO@CNTR*** hybrid sample after spray-coating of a NiO colloidal solution on CNTR, the NiO particle size was found to be slightly larger (Figure 2c,d) in comparison to the as-synthesized NiO QDs (as displayed in Figure S4d) because of agglomeration between the neighboring QDs (imperfection in the manual process of spray-coating) and/or embedment of ultrasmall particles beneath the entangled CNTs, which make them difficult to image through TEM.

### 2.3 Electrocatalytic Activities of Pristine (***CNTR***) and NiO QD-Coated (***NiO@CNTR***) Electrodes

#### 2.3.1 Hydrogen Evolution Reaction (HER) Activity

**Figure 3:**
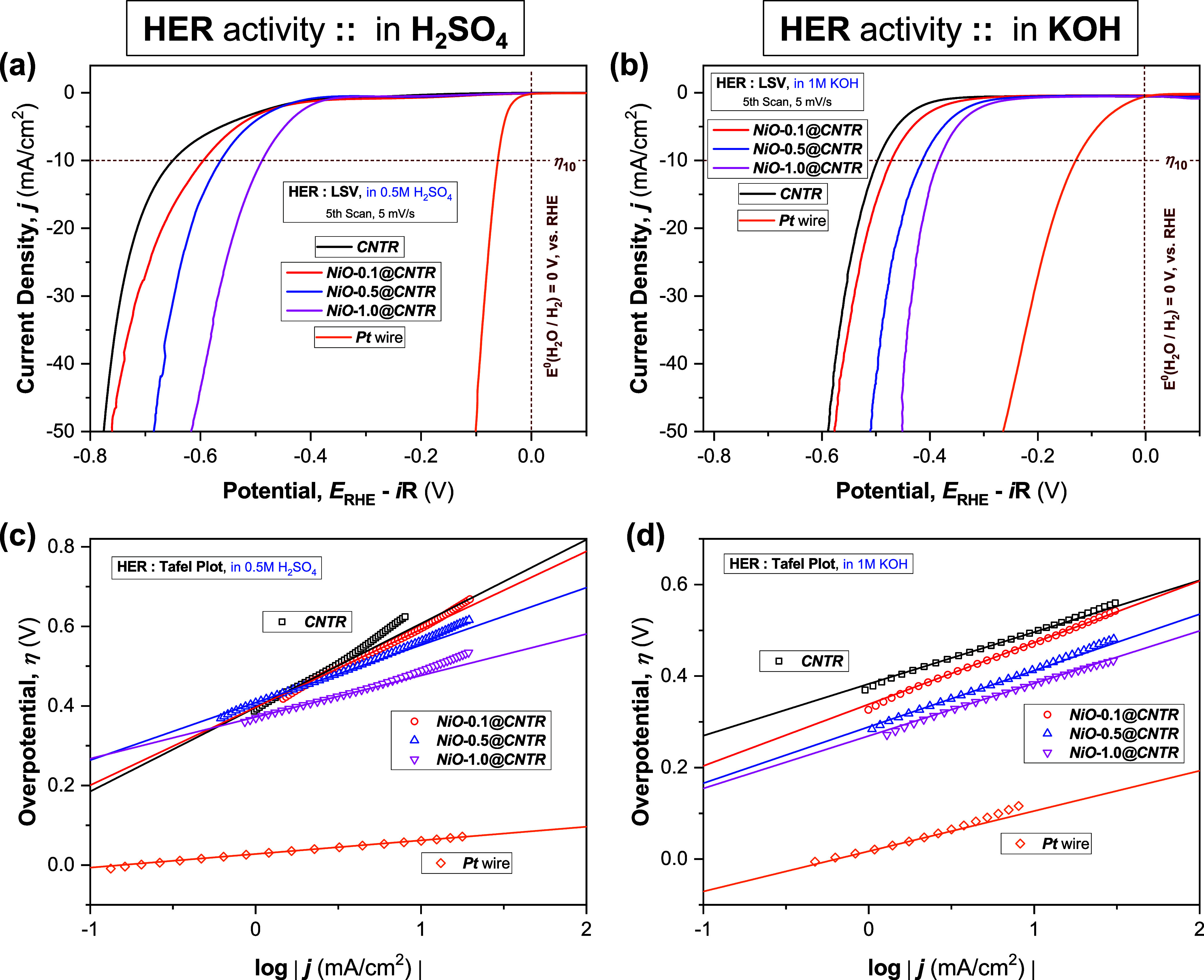
Electrocatalytic activity of pristine (***CNTR***) and NiO QD-coated (***NiO@CNTR***) electrodes. (a, b) Electrocatalytic activity for the hydrogen evolution reaction (HER) in N_2_-saturated aqueous (a) acidic (0.5 M H_2_SO_4_) and (b) alkaline (1 M KOH) media at a potential scan rate (ν) of 5 mV/s, without any rotation of the working electrode. (c, d) Corresponding Tafel plots of overpotential (η) vs log(*j*) measured in (c) 0.5 M H_2_SO_4_ and (d) 1 M KOH, where j represents the cathodic current density; all potentials were presented after *iR* correction. Respective data measured at the commercial ***Pt*** wire electrode are presented for comparison. Full details of the Tafel analysis and its associated calculations are described in the SI (Section S1, Analysis and Equations).

HER-catalytic performances of the laminated ***CNTR*** and ***NiO@CNTR*** electrodes were tested in N_2_-saturated acidic (0.5 M H_2_SO_4_, Figure 3a) and alkaline electrolytes (1 M KOH, Figure 3b). For comparison, the HER activities of a commercial ***Pt*** wire electrode were measured and are presented in Figure 3.

**Figure 4:**
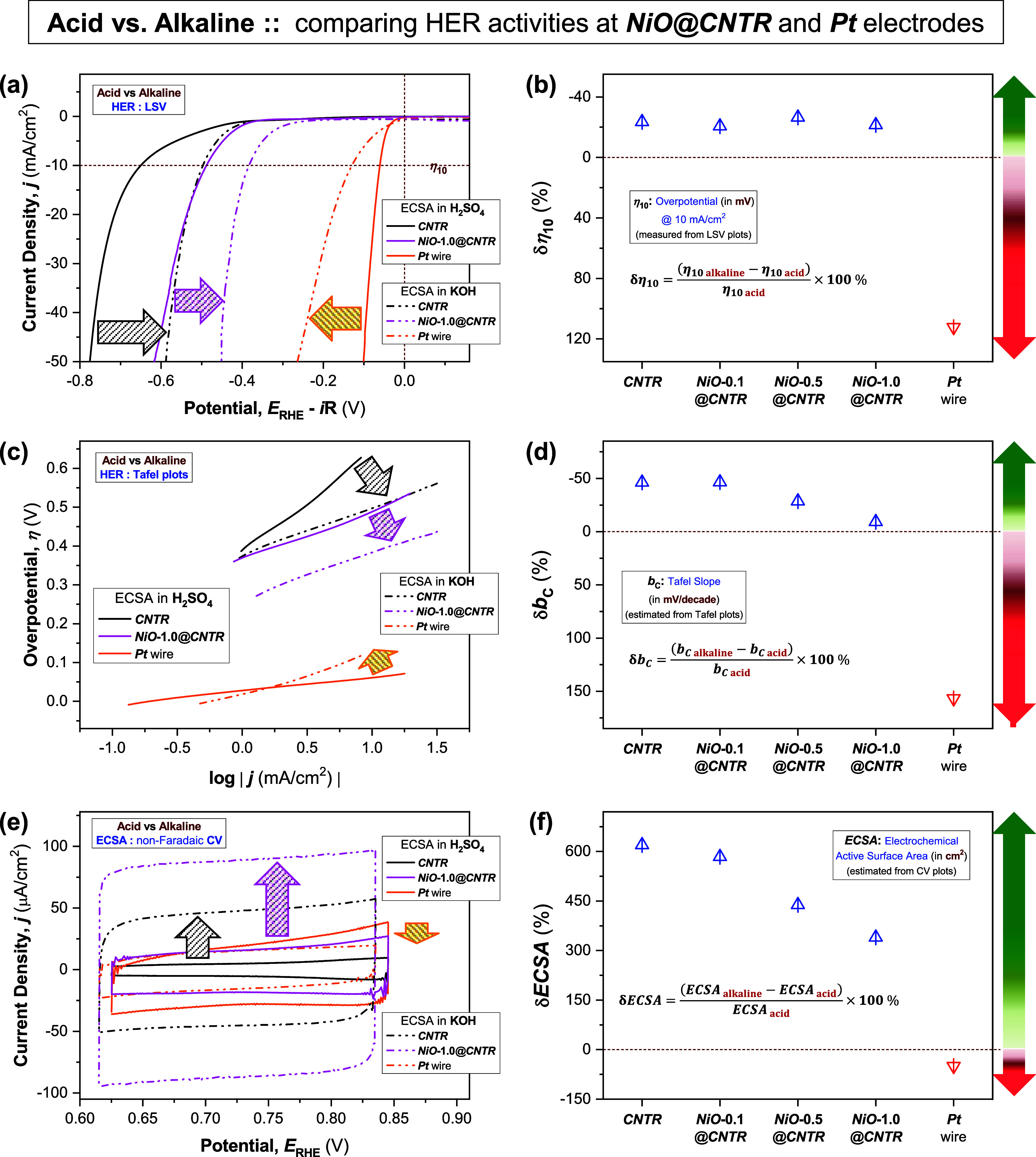
Acid vs alkaline electrolytes: HER activity. Comparison of HER performances of pristine (***CNTR***) and NiO QD-coated (***NiO*****-1.0*****@CNTR***) electrodes with a ***Pt*** wire in acidic (0.5 M H_2_SO_4_) and alkaline (1 M KOH) media: (a) LSV plots (*iR* corrected, at ν = 5 mV/s) and corresponding (b) δη_10_ values (change in η_10_, overpotentials @ 10 mA/cm^2^ value measured in alkaline media relative to that measured in acidic media). (c) Respective Tafel plots and corresponding (d) δ*b*_C_ values (change in *b*_C_, Tafel slopes). (e) CV plots performed in the non-Faradaic potential region (at ν = 50 mV/s) and (f) estimated δ*ECSA* values (change in *ECSA*, electrochemical active surface area). The respective HER parameters achieved at other electrodes with various NiO QD loadings are also presented in panels (b, d, f). The green and red arrows, drawn at the right-side boundaries of panels (b, d, f) exemplify the direction of the “improvement” and “decline” of the HER performance, respectively.

Three important observations follow from the *iR*-corrected polarization curves (linear sweep voltammograms, LSV plots, Figure 3a,3b):

(1) The pristine CNT ribbon electrode (***CNTR***, zero loading of NiO QDs) exhibited reasonable HER performances in both acidic (0.5 M H_2_SO_4_, Figure 3a) and alkaline (1 M KOH, Figure 3b) electrolytes. Herein, it should be mentioned that all of the laminated working electrodes were in a planar configuration and not subjected to any rotation (rotation speed of 0 rpm) during the electrochemical characterizations.

(2) With the loading of NiO QDs as electrocatalysts, the CNTR electrodes exhibited further improvement in HER performances in both acidic (Figure 3a) and alkaline (Figure 3b) electrolytes. The performance of ***NiO@CNTR*** electrodes was found to be enhanced with the increasing catalyst (NiO QDs) loading, irrespective of the electrolyte type, following the trend ***NiO*****-1.0*****@CNTR*** > ***NiO*****-0.5*****@CNTR*** > ***NiO*****-0.1*****@CNTR***.
(3) Most interestingly, comparing Figure 3a,b, it can be revealed that both ***CNTR*** and ***NiO@CNTR*** electrodes demonstrated a unique preference for the alkaline electrolyte, as evidenced by their improved HER activities obtained in a KOH solution compared to those measured in an acidic medium. For a clear picture, the HER performances of ***CNTR**,**NiO*****-1.0*****@CNTR*** (with the highest NiO QD loading, ***NiO*****-1.0**) electrodes, and the commercial ***Pt*** wire electrode are presented in Figure 4. Similar comparisons for other electrodes with varied NiO QD loadings are also demonstrated in Figure S5. As shown in Figure 4a, the LSV plots of the ***CNTR*** and ***NiO*****-1.0*****@CNTR*** electrodes in the alkaline electrolyte (1 M KOH) shifted further toward the theoretical HER potential, *E*^0^ (H_2_O/H_2_) = 0 V, exhibiting significantly improved catalytic activities compared to that achieved in an acidic medium (0.5 M H_2_SO_4_). In comparison, the polarization curve of the ***Pt*** wire electrode shifted to a higher potential in KOH relative to that measured in H_2_SO_4_, revealing degradation in HER activity from acid to alkaline electrolytes.

Detailed analysis of the LSV data, in terms of overpotential (η_10_, required to achieve a current density of 10 mA/cm^2^), disclosed that the η_10_ value of the ***CNTR*** electrode decreased by 24% (δη_10_, defined by the equation shown in Figure 4b) from η_10 acid_ = 649.4 mV (measured in acid, Table S2) to η_10 alkaline_ = 496.5 mV (in alkaline, Table S3). Similarly, all of the ***NiO@CNTR*** electrodes also exhibited negative δη_10_, as the acidic electrolyte was changed to alkaline. Notably, for the highest NiO QD loading (***NiO*****-1.0*****@CNTR*** electrode), the η_10_ reduced from η_10 acid_ = 488.1 mV (Table S2) to η_10 alkaline_ = 383.2 mV (Table S3), resulting in the negative δη_10_ (≈ −22%), as shown in Figure 4b. Negative δη_10_ is a measure of the “improvement” of HER activity (indicated by the green arrow drawn at the right boundary of Figure 4b). In contrast, positive δη_10_ quantifies the “deterioration” of HER performance (directed by the red arrow, Figure 4b). In contrast, for the ***Pt*** wire, the trend was opposite (Figure 4b), with the η_10_ value increased by 112%, resulting in the positive δη_10_ (Figure 4b), from 60.8 mV (in acid) to 129.1 mV (in alkaline), exhibiting sluggish HER activity of the ***Pt*** electrode in an alkaline electrolyte.

Furthermore, the Tafel analysis of the polarization curves, using eq S4, at low cathodic currents provided an important figure of merit, namely, the cathodic Tafel slope (*b*_C_), a valuable indicator for probing the rate-determining step of HER and could be estimated directly from the linear fitting of the Tafel plots (Figure 4c, also Figure 3c,3d). Comparing the performance in acid (Figure 3c) versus alkaline media (Figure 3d), for both pristine and NiO QD-coated CNTR electrodes, the *b*_C_ values are found to reduce in KOH (Table S3, *b*_C alkaline_ = 113 mV/decade for ***CNTR*** and 95 mV/decade for ***NiO*****-1.0*****@CNTR***) relative to that measured in H_2_SO_4_ (Table S2, *b*_C acid_ = 211 and 104 mV/decade, for ***CNTR*** and ***NiO*****-1.0*****@CNTR*** electrodes, respectively), leading to the negative values of δ*b*_C_ (46 and 9%, respectively), as observed in Figure 4d. Similar to the trend of δη_10_, a negative δ*b*_C_ represents faster and favorable HER kinetics, indicating an enhanced HER activity of the ***CNTR*** and ***NiO@CNTR*** electrodes in an alkaline medium. Predictably, the ***Pt*** wire electrode becomes a poor electrocatalyst exhibiting much higher *b*_C_ in KOH (87.9 mV/decade) than in H_2_SO_4_ (34.2 mV/decade), resulting in a positive δ*b*_C_ of over 150% (Figure 4d).

The enhancement in active site density is another critical indicator of a favorable HER activity, which could be quantified by estimating the effective electrochemically active surface area (*ECSA*) of the HER electrodes (eq S5), derived from the respective double-layer capacitance (*C*_dl_, eq S6) and measured via conducting cyclic voltammograms (CV) in a non-Faradaic potential region at various potential scan rates (ν) following McCrory et al.’s methodology.^48^Figure 4e represents a comparison between the CV plots (at ν = 50 mV/s) measured in acid and those performed in alkaline media for ***CNTR***, ***NiO*****-1.0*****@CNTR***, and the ***Pt*** wire. The detailed comparison of CV scans for all of the electrodes is shown in Figure S6a (acid electrolyte) and Figure S6b (alkaline electrolyte). The respective *C*_dl_ values were averaged from the slopes of the linear plots of cathodic (*i*_C_) and anodic current (*i*_A_) as the function of ν, measured in acid (Figure S6c) and alkaline (Figure S6d) media, respectively. Interestingly, all of the ***CNTR*** and ***NiO@CNTR*** electrodes exhibited a higher *C*_dl_, hence higher *ECSA* values in alkaline media (Table S3), compared to those achieved in an acidic electrolyte (Table S2). The calculated δ*ECSA* shows positive values for both ***CNTR*** (≈620%) and ***NiO@CNTR*** electrodes (≈340% at ***NiO*****-1.0*****@CNTR***), whereas it shows a negative value for the ***Pt*** wire electrode (δ*ECSA* ≈ −50%), as shown in Figure 4f. Opposite to the trends of δη_10_ and δ*b*_C_, the δ*ECSA* becomes positive for an enhanced HER activity, while a negative δ*ECSA* signifies a loss of HER activity due to a reduction in *ECSA.*

#### 2.3.2 Alkaline-Friendly Nature of CNTR and NiO@CNTR Electrodes

Such contrast between the CNTR electrodes (pristine and NiO QD-coated) and ***Pt*** electrodes can only be explained by the difference in HER kinetics and pathways in H_2_SO_4_ and KOH. As per the classical theory of cathodic HER,^49^ there are two possible reaction pathways that can be adopted, namely Volmer–Tafel (VT) and Volmer–Heyrovsky (VH). First, the adsorption of intermediate adsorbed hydrogen atoms (*H*_ads_) on the cathodic (working) electrode surface occurs via the Volmer reaction, through eq S1a in acidic or eq S1b in alkaline media.^33,50^ The next and final step would follow either the Heyrovsky desorption (via a reaction of *H*_ads_ intermediates with proton, eq S2a,b) or the Tafel desorption (via recombination of the two *H*_ads_, eq S3) to generate a H_2_ gas molecule.

The source of protons varies depending on the type of the electrolyte and plays a pivotal role in initiating the HER kinetics. In acidic media, the source of protons is H_3_O^+^, which directly initiates the HER (Volmer step, eq S1a). But in alkaline media, the source of protons is no longer H_3_O^+^ but simply H_2_O, the dissociation of which first takes place via eq S1b, producing the oxygen species (OH^–^) at the electrode/electrolyte interface.^33,50^

In acidic media, being a noble metal, the ***Pt*** electrode can easily facilitate the Tafel step (eq S3) directly after the Volmer step, following the VT pathways for HER, which is also evidenced by its *b*_C_ value close to 30 mV/decade (satisfying the condition for the Tafel reaction being the rate-determining step). However, in alkaline media, the production and adsorption of OH^–^ moieties at the Pt–electrolyte interface (eq S1b) impede the catalyzing capability^17^ of Pt toward the cleavage of the H–OH bond,^51^ decelerating the overall HER kinetics.^52^ Evidently, the *b*_C_ value of 87.9 mV/decade also suggested that the HER kinetics at the ***Pt*** wire electrode in KOH followed the VH reaction pathways^17^ (eq S1b followed by eq S2b).

On the contrary, in both acidic and alkaline media, the ***CNTR*** and ***NiO@CNTR*** electrodes followed the VH pathways, exhibiting high *b*_C_ (>40 mV/decade) values, with the Heyrovsky reaction as the rate-determining step. However, as observed in Figure 4d, in alkaline media, the *b*_C_ values are found to reduce substantially relative to those measured in acidic media, leading to the negative values of δ*b*_C_ and suggesting the escalation in the reaction rate and alkaline-friendly nature of ***CNTR*** and ***NiO@CNTR*** electrodes.

The OH^–^ moieties, associated with alkaline HER, interact favorably with the extended graphitic domains^34^ obtained from the hexagonal walls of CNT components of the ***CNTR***. Concurrently, the presence of inherent oxygen moieties (COOH, C–O, C=O and O–C=O, as evidenced by the XPS study, Figure 1f) on the ***CNTR*** surface favors the adsorption and dissociation of H_2_O molecules, efficiently initiating the HER process. Also, the same oxygen moieties could increase the supply of OH^–^ groups^33^ and other supportive H–OH species^34^ at the electrode/electrolyte interface, promoting the HER rate (Figure 4a,4b). In addition, our ***CNTR***, being a 3D ensemble of randomly oriented nanotubes, also provides a disordered carbon surface exposing an abundant density of catalytic sites,^33^ as evidenced by the significant enhancement of its *ECSA* value in KOH compared to that obtained in H_2_SO_4_ (Figure 4e).

As for the ***NiO@CNTR*** electrodes, it can be suggested that NiO QDs and CNTR would work in concert, where the oxophilic power of NiO QDs and existing oxygen moieties on the CNTR surface favor the adsorption and dissociation of H_2_O molecules. At the same time, synergistically, the adjacent C atoms (the graphitic domains) provide the active sites for the enhanced recombination of *H*_ads_ intermediates.^5,6,13,51,53^ Here, the “synergistic coupling” arises from the good adhesion with an improved interface between the NiO QDs and CNTR surface, which works in complement to each other for maintaining the uninterrupted chemical reaction steps, accelerating the preferred catalytic reactions and leading to a desired long-term stability in the catalytic performances. Such a “synergistic effect”^5,6^ has also been reported in earlier studies involving hybrid electrocatalysts, such as NiO electrocatalysts on metallic support^53^ or Ni/NiO catalysts.^13,51^

#### 2.3.3 Oxygen Evolution Reaction (OER) and Bifunctional Activity

**Figure 5:**
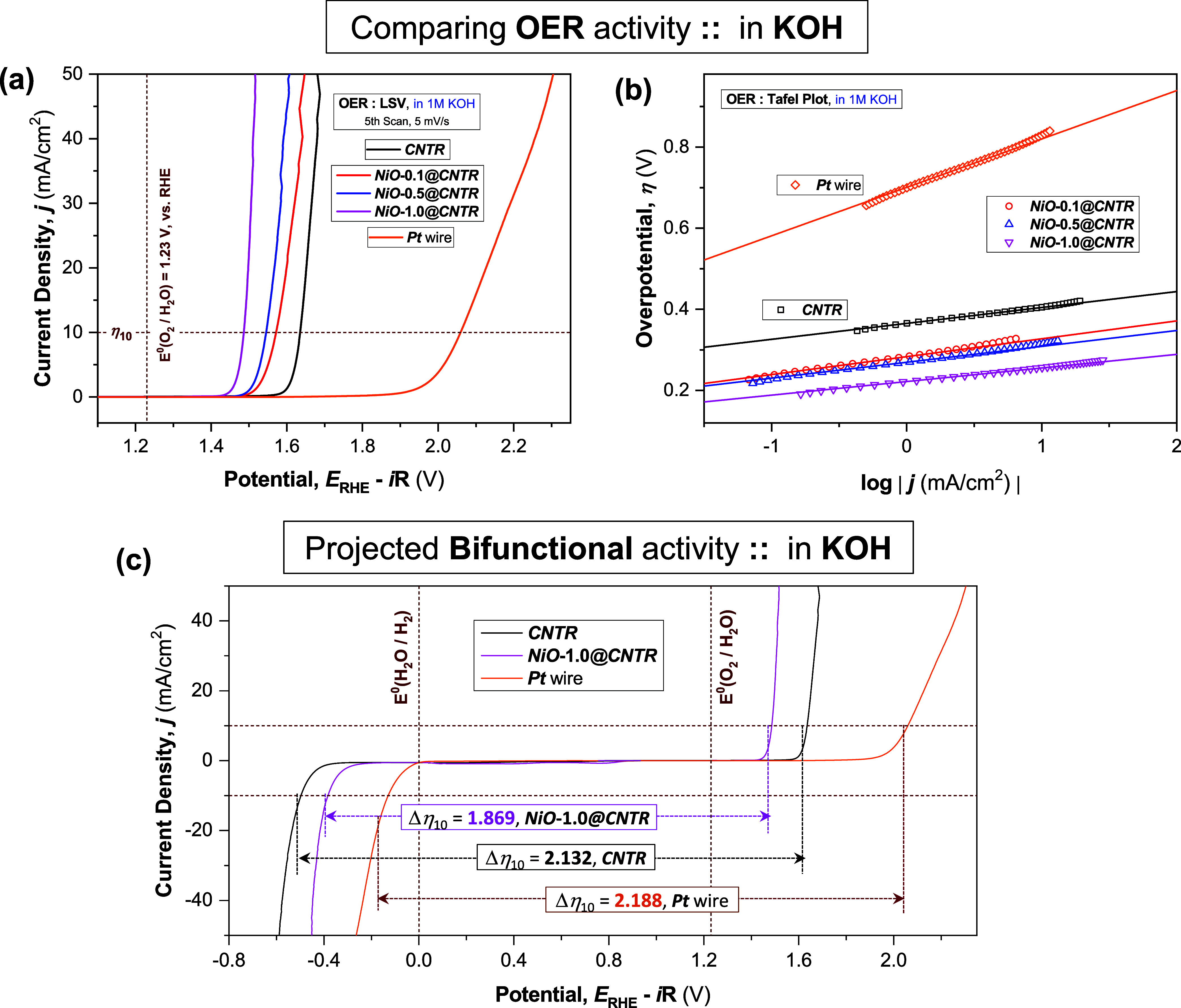
Oxygen evolution reaction (OER) and bifunctional activity. (a) OER activity of ***CNTR*** and ***NiO@CNTR*** electrodes in a N_2_-saturated alkaline medium (1 M KOH), at ν = 5 mV/s, without any rotation of the working electrode. (b) Corresponding Tafel plots of overpotential (η) vs log(*j*) measured in 1 M KOH; all potentials were presented after *iR* correction. Respective data measured at the commercial ***Pt*** wire electrode are presented for comparison. (c) Projected bifunctional activity of ***CNTR*** and ***NiO*****-1.0*****@CNTR*** electrodes: values of full-cell potential Δη_10_ estimated from the difference between overpotential values (η_10_, estimated at ±10 mA/cm^2^) achieved from the respective HER and OER polarization curves (recorded in 1 M KOH), with the ***Pt*** wire as a reference for comparison.

To realize the bifunctional nature of the proposed electrodes being appropriate for the overall water-splitting application, the OER performances of the ***CNTR*** and ***NiO@CNTR*** electrodes were also evaluated in N_2_-saturated 1.0 m KOH (Figure 5a). The ***CNTR***, without any additional catalysts loading, displayed a substantially superior performance (η_10_ ≈ 1.635 mV and *b*_A_ ≈ 39.2 mV/decade) relative to the ***Pt*** wire electrode (η_10_ ≈ 2.059 mV and *b*_A_ ≈ 119.4 mV/decade, Table S4). With the loading of NiO QDs, the ***NiO@CNTR*** electrodes exhibited further improvement in OER performances, lowering both η_10_ and *b*_A_ (anodic Tafel slope, Figure 5b) values as the NiO QD loading increases. The ***NiO*****-1.0*****@CNTR*** electrode exhibited the best catalytic activities of O_2_ generation, with η_10_ of 1.486 mV and *b*_A_ of 33.5 mV/decade in an alkaline electrolyte (1 M KOH, Table S4).

Compared to HER, the OER mechanism is comparatively more complicated and less clarified in the literature.^52,54^ In a simple view, OER follows a 4e^–^ pathway (eqs S8–S11) in alkaline media, initiated with the hydroxide (OH^–^) anion and associated with the adsorption of *O*_ads_ intermediates. Hence, based on the earlier arguments for the alkaline-HER activity, it can be reasonably anticipated that the ***Pt*** wire electrode will suffer from sluggish OER activity. In contrast, both the ***CNTR*** and ***NiO@CNTR*** surfaces could readily favor the adsorption of both OH^–^ and *O*_ads_ intermediates, accelerating the subsequent reaction steps and displaying a significantly superior OER activity compared to the ***Pt*** counterpart.

On the basis of the above-mentioned studies, it can be rationally predicted that both ***CNTR*** and ***NiO@CNTR*** electrodes would be excellent bifunctional catalysts for HER and OER in alkaline media. As shown in Figure 5c, replotting both cathodic (HER) and anodic (OER) polarization curves, collected by three-electrode configuration, the potential difference (Δη_10_) between HER and OER current density (of 10 mA/cm^2^) for ***CNTR***, ***NiO*****-1.0*****@CNTR***, and ***Pt*** wire electrodes represent an expected full-cell potential window. The calculated values of full-cell potential Δη_10_, the difference between the overpotential values (at ±10 mA/cm^2^) achieved from the relevant HER and OER polarization curves, are 1.869, 2.132, and 2.188 V, for ***NiO*****-1.0*****@CNTR***, ***CNTR***, and ***Pt*** wire, respectively. These values clearly signify the superior performance of ***NiO*****-1.0*****@CNTR*** compared to that of the ***Pt*** wire, suggesting its potential application for a practical overall water-splitting (OWS) device in an alkaline electrolyte, employing the same electrode materials as both the anode and the cathode.

### 2.4 Bifunctional Catalytic Performance for Alkaline Overall Water Splitting (OWS)

**Figure 6:**
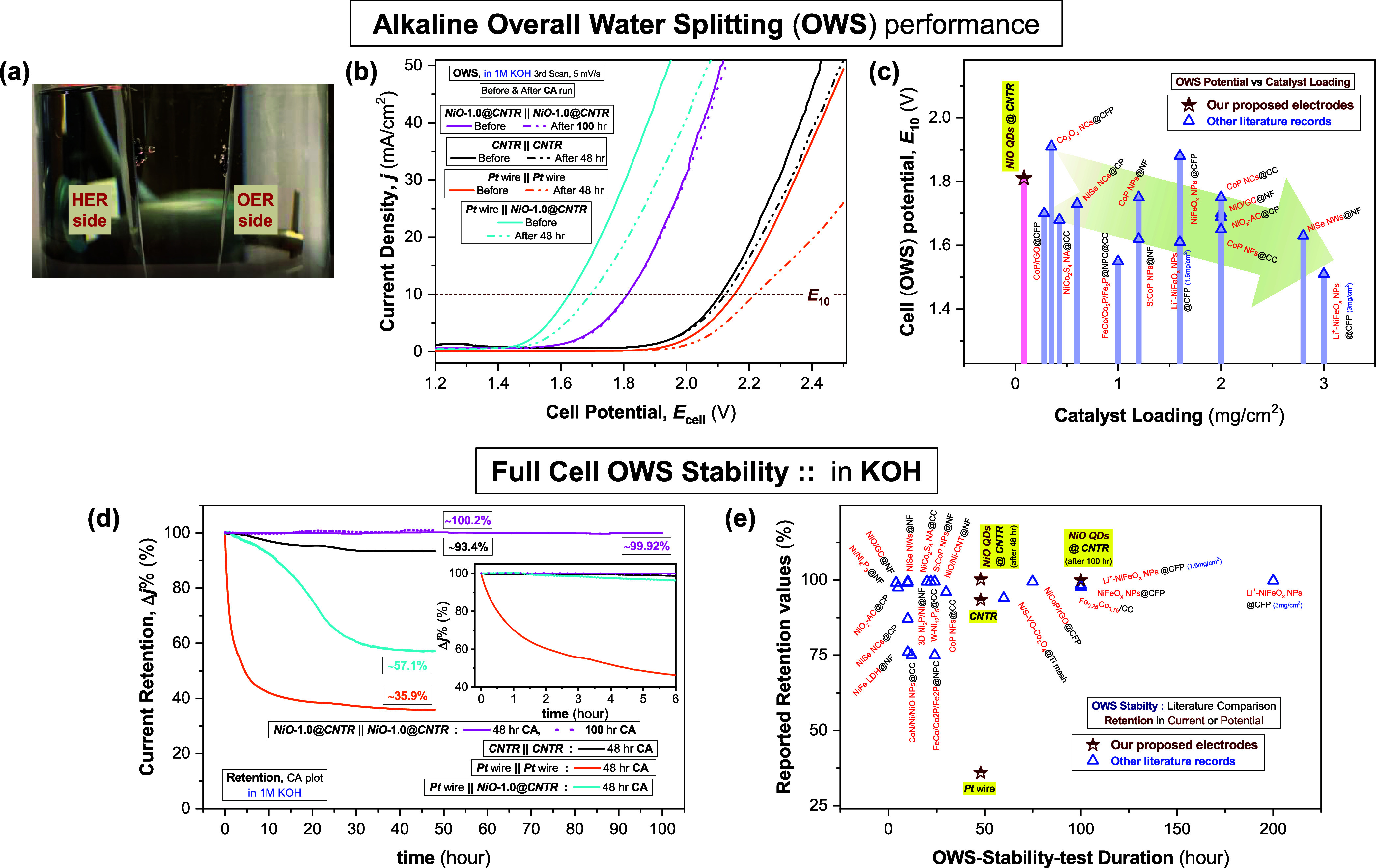
Bifunctional catalytic activity of ***CNTR*** and ***NiO*-1.0*@CNTR*** electrodes using the same electrode materials as both the anode and the cathode in a two-electrode (2E) alkaline electrolyzer cell. (a) Digital image of the experimental setup during the overall water-splitting (OWS) performances (of the ***NiO*****-1.0*****@CNTR*****||*****NiO*****-1.0*****@CNTR*** electrolyzer) in a N_2_-saturated alkaline medium (1 M KOH). (b) Polarization (OWS, without *iR* correction) plots for ***CNTR*****||*****CNTR*** and ***NiO*****-1.0*****@CNTR*****||*****NiO*****-1.0*****@CNTR*** electrolyzers with the ***Pt*** wire **||*****Pt*** wire as a reference for comparison; at ν = 5 mV/s: comparison of LSV plots measured before and after the stability test (presented in panel (d)). For comparison, an asymmetric cell configuration (***Pt*** wire **||*****NiO*****-1.0*****@CNTR***) was also presented, using the ***Pt*** wire as a cathode and ***NiO*****-1.0*****@CNTR*** as an anode. (c) Brief literature comparison of the reported OWS cell potential (*E*_10_) as a function of the respective catalyst loading, as reported for various bifunctional electrocatalysts in 1 M alkaline electrolytes (KOH or NaOH). (d) Long-term stability of OWS (chronoamperometry, CA), monitoring the current retention at the respective *E*_10_ (recorded at a current density of 10 mA/cm^2^ from the initial OWS plots (before CA measurement, panel (b))). The inset represents the respective current retention during the first 6 h. All of the electrolyzers were subjected to a 48 h CA study. Only, for our proposed electrodes, the performance of the ***NiO*****-1.0*****@CNTR*****||*****NiO*****-1.0*****@CNTR*** electrolyzer was repeated (using a fresh set of electrodes) for a more extended stability study for 100 h. (e) Brief literature comparison of OWS stability performance of various bifunctional electrocatalysts reported in 1 M alkaline electrolytes (KOH or NaOH), displaying the reported current (or potential) retention values in the stability-test duration scale.

The OWS performance was assessed by assembling the same electrode materials as both the anode and the cathode in a two-electrode (2E) alkaline electrolyzer cell (Figure 6a, also find the Supporting Video SV2_OWS). In Figure 6b, the polarization curve (without *iR* correction) of the ***NiO*****-1.0*****@CNTR*****||*****NiO*****-1.0*****@CNTR*** electrolyzer exhibits a cell potential (*E*_10_) of ∼1.81 V (recorded at a current density of 10 mA/cm^2^), significantly better than that obtained with the ***Pt*** wire **||*****Pt*** wire electrolyzer. Interestingly, the ***CNTR*****||*****CNTR*** electrolyzer also exhibited an excellent OWS performance with an *E*_10_ of 2.11 V, better than that of the ***Pt*** wire **||*****Pt*** wire electrolyzer (*E*_10_ ≈ 2.15 V). In this study, we employed the OWS performance of the ***Pt*** wire only as a “reference” for the comparative study with our proposed electrodes, as it is commonly used for comparison and sometime considered as a benchmark material for such catalysis application specifically for HER. The water-splitting potential (*E*_10_) values obtained from the 2E electrolyzer (in symmetric cell configuration) are lower than the predicted full-cell potential Δη_10_ values calculated, as shown in Figure 5c, which imply benefits from the effective integration of OWS devices using the same electrode materials as both the anode and the cathode. For comparison, an asymmetric cell configuration (***Pt*** wire **||*****NiO*****-1.0*****@CNTR***) was also presented, using the ***Pt*** wire as a cathode and ***NiO*****-1.0*****@CNTR*** as an anode, which exhibited excellent OWS performance with an *E*_10_ ≈ 1.63 V (Figure 6b), slightly higher than the predicted Δη_10_ value obtained, as shown in Figure 5c. However, as observed and discussed later, such asymmetric cell configuration cannot provide adequate stability in long-term OWS performance because of the degradation of Pt metal in an alkaline medium.

As compared with other materials available in the literature (see Table S5, in SI), our bifunctional ***NiO*****-1.0*****@CNTR*** electrode exhibited a promising OWS potential (*E*_10_ of ∼1.81 V), with the minimal loading of the NiO QD catalyst (≈83 μg/cm^2^ only), which is comparable to the *E*_10_ values reported in other single (transition) metal-based bifunctional catalysts with at least 1 order of magnitude higher “catalyst loading”,^10,14,15,44−46,55−61^ such as NiO nanosheets (NSs) on a nickel foam or NF (*E*_3_ > 1.8 V with a loading of 0.53 mg/cm^2^),^58^ α-NiOOH NSs on NF (*E*_4_ > 1.75 V with 0.74 mg/cm^2^ loading),^58^ NiO/GC on NF (*E*_10_ ∼ 1.7 V with 2 mg/cm^2^ loading),^59^ NiO_*x*_-AC on carbon paper or CP (*E*_10_ ∼ 1.69 V with 2 mg/cm^2^ loading),^60^ Ni(OH)_2_ NSs on NF (*E*_10_ ∼ 1.82 V),^15^ Co_3_O_4_ nanocrystals (NCs) on carbon fiber paper or CFP (1.91 V with 0.35 mg/cm^2^ loading),^46^ Ni_0.5_Se||Ni_0.75_Se NCs on CP (1.73 V with 0.6 mg/cm^2^ loading),^44^ W-Ni_12_P_5_ on carbon cloth or CC (1.73 V),^61^ CoP NCs on CC (1.75 V with 2 mg/cm^2^ loading),^55^ and CoP NPs on NF (1.75 V with ∼1.2 mg/cm^2^ loading).^14^Figure 6c presents a graphical comparison of the “*E*_10_” values for various bifunctional electrocatalysts reported in the past literature as a function of the respective “catalyst loading”. The literature comparison (Table S5 and Figure 6c) also presents several reports of lower *E*_10_ values, which are mainly reported for the bimetallic or hybrid catalyst systems^13−16,43,47,62−66^ and the catalysts with much higher loading compared to our work;^10,13,16,47,55^ for example, NiFe layered double hydroxides on NF (1.7 V),^15^ S-doped CoP NPs on NF (1.62 V with ∼1.2 mg/cm^2^ loading),^14^ CoN/Ni/NiO NPs on CC (1.56 V),^63^ Fe_0.25_Co_0.75_ on CC (1.66 V),^64^ NiCo_2_O_4_ nanowires (NWs) and NiCo_2_S_4_ NW-array on carbon cloth (1.98 and 1.68 V, respectively, with 0.43 mg/cm^2^ loading),^43^ NiFeO_*x*_ and Li^+^-NiFeO_*x*_ nanoparticles on carbon fiber paper or CFP (1.88 and 1.61 V, respectively, with ≈1.6 mg/cm^2^ loading),^16^ 3D Ni_2_P/Ni on NF (1.49 V),^65^ NiCoP/rGO on CFP (1.59 V with ∼0.15 mg/cm^2^ loading),^66^ NiO/Ni-CNT||NiFe LDH on NF (1.41 V with ∼8 mg/cm^2^ loading),^13^ or Ni/Ni_8_P_3_ on NF (1.61 V with ∼10.5 mg/cm^2^ loading).^47^

While comparing the performance of our ***NiO@CNTR*** electrode with the literature, it is also worth emphasizing the simple, rapid, and scalable fabrication process (spray-coating) of our proposed electrode. Core catalysts, the PiNE-synthesized NiO QD colloids have the advantage of a single-step synthesis process with no further purification needed; also, the particle size remains stable for a long time (years), and no agglomeration happens with no surfactant needed. At the same time, from the viewpoint of the CNTR matrix, there is no length limitation in producing CNTR as long as the precursor feed is available and the material remains mechanically and chemically stable without any need for specific storage. Moreover, the macroscopic form of CNTR is easily formable to various shapes and sizes, which is an added advantage for customized fabrication of electrodes. This gives a competitive advantage to our electrode for easy transfer of this technology to an industrial scale for practical applications.

The stability or durability of the electrodes was assessed by using chronoamperometry (CA, Figure 6d), maintaining the respective cell potential (*E*_10_, recorded at a current density at 10 mA/cm^2^ from the initial OWS plot, Figure 6b). Impressively, the ***NiO*****-1.0*****@CNTR*****||*****NiO*****-1.0*****@CNTR*** electrolyzer reported in this study is found to retain the initial current density even after 4 days, which shows the current retention of ≈100.2% after 48 h and ≈99.92% after 100 h of electrolysis operation, as shown in Figure 6d, suggesting a promising performance of the ***NiO@CNTR*** electrode for a prolonged OWS run. Interestingly, the ***CNTR*****||*****CNTR*** electrolyzer also exhibited high stability in current (losing only ∼6% of the initial value) over 48 h of continuous run (Figure 6d). Relatively, the ***Pt*** wire **||*****Pt*** wire electrolyzer retained only 36% of the initial current after the stability test of 48 h (Figure 6d). Furthermore, we tested the reproducibility of our results by repeating the stability test for the ***Pt*** wire||***Pt*** wire and ***CNTR***||***CNTR*** electrolyzers for two different sets of electrodes, as shown in Supporting Figure S7. Notably, as mentioned earlier, the ***Pt*** wire **||*****NiO*****-1.0*****@CNTR*** asymmetric electrolyzer also failed to maintain reasonable stability, retaining only ∼57% of the initial current density after 48 h run. These findings would justify our quest for a “bifunctional” electrode that can catalyze both HER and OER, leading to a simple and symmetric cell configuration with stable and effective OWS performance. Besides, configuring the electrolyzer cell with the same materials for both electrodes (cathode and anode) would help to avoid the complexity of using different electrodes for HER and OER and unwanted “contamination” of electrodes and electrolytes. A comparative study^10,13−16,43,44,47,55,56,59−66^ of current (or potential) retention over time, as reported in a previous literature, is shown in Figure 6e, which indicates the superior nature of our electrodes in terms of long-standing stability for OWS application.

“Long-term” stability in the current acquired with slight reduction and subsequent growth cycles implies a self-healing nature of the catalysts, originating from the “synergistic coupling”^5,6^ between NiO QDs and the CNTR matrix. Earlier reports^16^ justified such “long-standing stability” as an outcome of the “slow” adsorption/desorption kinetics of the reaction intermediates that happened at the catalyst’s sites (e.g., NiO QDs). With time, as the reaction proceeds, the adsorbed intermediates impede the electrolyte diffusion to the catalyst sites, slowing down the next reaction steps. However, the slow kinetics would provide sufficient time for the neighboring active sites (e.g., C or Ni^0^) to facilitate the desorption step, consequently refreshing the surfaces and boundaries of the interconnected particles.^16^ Such a course of “self-healing” action would, in turn, recover the catalytic activity, regaining the current to its initial value.^5,6^ In addition, the “slow” formation of gas bubbles leads to a gradual growth of bubbles adhered to the electrodes’ surface (please find the Supporting Video SV2_OWS displaying the phenomenon during the OWS run), hindering effective contact with the electrolyte and resulting in a decline of reaction. As the bubbles grow to a limit, sudden release of bubbles (H_2_ and O_2_ gases) from the surface of electrodes (cathode and anode, respectively) would occur in periodic intervals, which could also help remove surface residues and contribute to the undulating activation process observed.^13,16^

The LSV measurements before and after the stability (CA) test (Figure 6b) reveal that our proposed ***NiO*****-1.0*****@CNTR*****||*****NiO*****-1.0*****@CNTR*** electrolyzer exhibited a repetitive LSV plot maintaining the initial OWS performance even after 100 h of run, as evidenced in Figure 6b, suggesting a favorable utilization of the ***NiO@CNTR*** electrode for a long-term OWS application. Interestingly, the pristine ***CNTR*****||*****CNTR*** electrolyzer exhibited a minor degradation in OWS performance after a 48 h stability test. Comparatively, the LSV performances of the ***Pt*** wire **||*****Pt*** wire and ***Pt*** wire **||*****NiO*****-1.0*****@CNTR*** electrolyzers suffered heavily after the stability test of 48 h (Figure 6b).

## 3 Conclusions

This study successfully demonstrates the bifunctional electrocatalytic activity of a novel electrode, ***NiO@CNTR***, by hybridizing macroscopically assembled carbon nanotube ribbons (CNTRs, directly spinning from the CVD reactor) and NiO QDs (synthesized by PiNE) for efficient and highly stable alkaline-based OWS applications. The comparison of HER activities in different electrolytes revealed that the proposed electrodes strongly prefer the alkaline media over the acidic one, as evidenced by the lowering of η_10_ and *b*_C_ parameters and enhancement of *ECSA*. In contrast, the noble metal catalyst (here, a ***Pt*** wire electrode) exhibited a considerable degradation in HER activity in an alkaline electrolyte, as substantiated by the increase in η_10_ and *b*_C_ and the decrease in *ECSA*, compared with its acidic HER. Collectively, in a 2E configuration for alkaline electrolysis, by assembling the same electrode materials as both the anode and the cathode for HER and OER, superior performance of ***NiO@CNTR*** relative to ***Pt*** was observed. Furthermore, this superior performance of our proposed ***NiO@CNTR*** electrode has been achieved with a very low NiO QD loading (only 83 μg/cm^2^) compared to other loading values reported in the literature for various bifunctional electrocatalysts. ***NiO@CNTR*** exhibited a cell potential of only 1.81 V at 10 mA/cm^2^ for OWS, which is much lower than that of the ***Pt*** wire (*E*_10_ ≈ 2.15 V). It also demonstrated remarkable catalytic activity accompanied by “long-term” OWS stability, retaining ∼100% of the initial current after a 100 h long OWS run. The superior performance of ***NiO@CNTR*** is mainly attributed to the alkaline-friendly and bifunctional nature of both the NiO QDs and the CNTR support as well as the synergistic coupling between them. This proposed ***NiO@CNTR*** electrode with high chemical and mechanical stability can provide a viable solution for fuel cell technology as the material synthesis and electrode fabrication process involve simple and scalable techniques by avoiding any requirement of a surfactant, a binder, and complex purification steps. This study also shows a pathway toward cost-effective and large-scale sustainable solutions for electrochemical water-splitting devices, which can be used as an alternative energy source with limited environmental effects.

## 4 Experimental Section

### 4.1 Chemicals

All analytical grade chemicals were purchased and used without any further purification process. Ferrocene (Fe(C_5_H_5_)_2_, ≥98%), thiophene (C_4_H_4_S, ≥99%), sulfuric acid (H_2_SO_4_, 95–98%), and potassium hydroxide (KOH, ≥90%, flakes) were purchased from Sigma-Aldrich. Pure methane and hydrogen gases (BOC, UK) were used as the reactant gas during the growth of CNTR by the thermal CVD technique. A Ni foil of 99.5% purity (GoodFellow) was used as an anode as well as the Ni source in the PiNE synthesis. Commercial Pt wires (99.95% purity) were purchased from BASi and used as counter electrodes for HER and OER tests. Milli-Q water, with a resistivity of ≈15 MΩ/cm, was used to prepare all solutions (PureLab option, UK).

### 4.2 Synthesis of CNT Ribbons (CNTRs)

CNTRs were synthesized using a floating catalyst-assisted thermal CVD technique.^20,22,23^ For the synthesis, methane was used as a carbon precursor, and an iron (Fe) floating catalyst was obtained by decomposing ferrocene at high temperature. At a temperature above 400 °C, ferrocene starts to break down and releases Fe atoms. The aggregation of Fe atoms eventually leads to the formation of Fe nanoparticles (NPs), which serve as catalytic sites for the growth of CNTs. Hydrogen was used as a promoter/carrier gas during the CVD synthesis of the CNTs. Carbon atoms rearrange to form CNTs on the surface of catalyst NPs, collectively forming a macroscopically assembled CNT network, which can be extracted from the furnace onto a moving winder (Figure S1a,b).

Following our earlier publication by Brunet et al.,^20^ the synthesis of the CNT aerogel was performed under a methane flow rate of 160 sccm and a carrier hydrogen flow rate of 1350 sccm through a tube furnace held at 1290 °C. For the formation of Fe NP catalysts, the ferrocene and thiophene supplies were maintained by a carrier H_2_ flow with rates of 130 and 90 sccm, respectively. Figure S1c,d represents a typical as-synthesized 3D ensemble of a randomly oriented CNT network (also Figure 1b).

Prior to any characterization or application, the as-synthesized macroscopic CNT network was further processed by pressing it between two glass microscope slides for a few (3–5) minutes under an applied force of ≈18 N to form a flat and compact fabric-like material (Figure 1c,d). Supporting Figure S1e–k reveals the flexible and lightweight nature of a typical CNTR (also see the Supporting Video SV1_CNTR). Depending on the compressive force applied, the thickness of the CNTR sample can be tailored. In this study, the CNTR thickness was maintained between 3 and 5 μm (Figure 1e). The material (CNTR) and its synthesis process were extensively characterized in our previous publication.^27^

### 4.3 Synthesis of NiO Quantum Dots (QDs)

NiO QDs with a narrow size distribution were synthesized using PiNE,^26,35−37^ as demonstrated in the digital image (Figure S4a) and the schematic diagram (Figure S4b) of the experimental setup. Similar to our earlier publication by Chakrabarti et al.,^26^ a nickel foil of a 99.5% purity (Goodfellow Cambridge Ltd.) was used as an anode as well as the Ni source in the synthesis. A nickel tube (0.7 mm internal diameter and 1 mm outer diameter) was used as the cathode; the distance between the anode and the cathode was kept constant at about 1.8 cm. Pure He gas was flown at a flow rate of 50 sccm through the nickel tube, and an initial voltage of 3 kV was used to initiate the plasma between the end of the nickel tube and the surface of the ethanol. For each synthesis batch, 15 mL of ethanol was used, the immersed area of the Ni foil was maintained at 1.5 cm × 1.5 cm, and the distance between the nickel tube and the surface of the liquid was kept at 2 mm.

For igniting the plasma, a direct current (DC) voltage of 3 kV was applied and set until the current reached 5 mA. Then, the current was maintained constant (around 5 mA) by gradually decreasing the voltage from 3 to 2 kV. A total deposition time of 45 min was retained for each synthesis of NiO QDs with an intermediate pause of 2 min at each 10 min interval.

### 4.4 Deposition of NiO QDs on CNTR

For fabrication of NiO QD-coated CNTR electrodes (***NiO@CNTR***), the PiNE-synthesized NiO QD-ethanol colloids were spray-coated onto the laminated CNTR electrodes at three different volume concentrations, roughly 66 μg/mL (as-synthesized colloids, symbolized as *NiO*-1.0), ≈33 μg/mL (two times diluted, *NiO*-0.5), and ≈6.6 μg/mL (diluted by ten times, *NiO*-0.1). Detailed spray-coating parameters and NiO QD loading concentration are given in Table S1. In summary, three ***NiO@CNTR*** electrodes, namely ***NiO*****-1.0*****@CNTR***, ***NiO*****-0.5*****@CNTR***, and ***NiO*****-0.1*****@CNTR***, were loaded by 83 μg/cm^2^, 42 μg/cm^2^, and 8.3 μg/cm^2^ of NiO QDs, respectively. For comparison, the pristine CNT ribbon was also employed as an electrode (***CNTR***), representing its electrocatalytic activity without any additional catalysts.

### 4.5 Fabrication of Laminated Electrodes of Pristine and NiO QD-Coated CNTR

For electrode preparation, the as-synthesized CNT ribbon (Figure 1d) was cut into desired pieces, and those CNTR pieces were manually interlaced with adhesive copper tape and nickel conducting paste. The Cu tape (and Ni conducting paste) is used only to extend the electrical contact for the current collection. Finally, the assembly was laminated, maintaining a window (only on one side of the laminate) for the exposure of CNTR (only) to the electrolyte. The circular window represents the active area (0.45 cm diameter) of our laminated electrodes for electrochemical reaction, as shown in Figure S3a.

### 4.6 Material Characterizations and Instrumentation

SEM images of the samples were obtained with a JEOL JSM-6010PLUS operating at a 20 kV acceleration voltage. For the TEM measurements. the JEOL JEM-2100F system equipped with a field-emission electron gun operated at 200 kV was used. Energy dispersive spectroscopic (EDS) measurements were carried out using an OXFORD 80 mm^2^ X-Max Silicon drift detector fitted onto the TEM. The software from INCA was used to analyze the data. EELS measurements were carried out in the scanning TEM mode using the integrated GATAN Enfinium spectrometer. X-ray photoelectron spectroscopy (XPS) was used to analyze the chemical composition of the materials. An X-ray source (Al = 1486 eV) with a spot size of 400 μm^2^ was used with a Kratos Axis Ultra DLD spectrometer. The sample analysis chamber pressure was maintained at 10^–9^ bar for all measurements. During the measurements, the operating current and voltage were 10 mA and 15 kV, respectively. Specific region scans were performed at a resolution of 0.05 eV and a pass energy of 20 eV. The obtained spectra were calibrated using the C 1s peak located at 284.5 eV. Raman spectroscopy was used to acquire experimental information about the electronic and vibrational properties of the CNTs and was carried out using a Horiba Labram 300 Raman spectrometer, using a HeNe (633 nm) laser for excitation.

Prior to any basic characterizations (SEM, Raman and XPS studies), a tiny piece of CNTR was laid down onto a silicon substrate. Several drops of ethanol were applied and evaporated at an elevated temperature, resulting in a dry ribbon that adhered to the silicon substrate. TEM samples of CNTR samples were prepared using a minute piece of CNTR (collected by tweezers) on a holey carbon-coated copper grid of 300 meshes, followed by dropping a few microlitres of ethanol and subsequently drying overnight. For the NiO QDS, diluted ethanol-dispersed NiO QDs were drop-cast on a silicon substrate and dried under an elevated temperature before SEM and XPS studies. TEM samples were prepared by dropping 1 μL of ethanol-dispersed NiO QDs on a holey lacey carbon-coated (300 mesh) copper grid and dried under incandescent lamp light radiation for 5 min. Then, the grid was kept at room temperature for a few hours with a glass protection cover.

#### 4.6.1 Electrochemical Studies

All electrochemical studies were carried out using a BioLogic potentiostat, SP-200 (Bio-Logic Science Instruments Ltd., UK), coupled with an EIS channel. A typical three-electrode system (Figure S3b) was used to examine the activity of the proposed pristine ***CNTR*** and ***NiO@CNTR*** laminated electrodes (Figure S3a), used as the working electrode. A platinum wire (99.95% purity, BASi) and Ag/AgCl (3 M KCl, BASi) were used as counter (CE) and reference electrodes (RE), respectively. In addition, a commercial Pt wire (99.95%, BASi) was also purchased from BASi and used as a reference working electrode for HER and OER tests.

Two (aqueous) electrolyte solutions, 0.5 M H_2_SO_4_ (pH = 0.29) and 1 M KOH (pH = 14.0), were freshly prepared and subjected to the N_2_ gas purging for hours to remove the dissolved oxygen. Before conducting the HER or OER activity studies, all of the working electrodes were preconditioned by cyclic voltammetry (CV) at a potential scan rate (ν) of 100 mV/s for at least 20 scans.

The polarization studies were carried out by employing linear sweep voltammetry (LSV) at ν of 5 mV/s. Herein, it should be mentioned that all of the laminated working electrodes were in a planar configuration, not subjected to any rotation (rotation speed of 0 rpm). All of the electrochemical experiments were conducted at room temperature (20 ± 2 °C).

All of the measured potentials were converted to a reversible hydrogen electrode (RHE) using the Nernst equation: *E*_RHE_ = *E*_Ag/AgCl_ + (0.059 × pH) + *E*_Ag/AgCl_^0^ (where, *E*_Ag/AgCl_^0^ = 0.210 V at 25 °C). The pH values of 0.5 M H_2_SO_4_ and 1 M KOH are estimated as 0.29 and 14.0, respectively. Furthermore, all of the potentials in the reported cathodic (HER) or anodic (OER) polarization curves (LSV plots) were corrected for ohmic losses (*iR* drop correction) manually by using AC-impedance solution (or electrolyte) resistance (*R*_s_).

The double-layer capacitance (*C*_dl_) was estimated by performing the CV in the non-Faradaic potential region at different scan rates (ν = 10, 20, 30, 40, 50, and 60 mV/s). The *C*_dl_ value was averaged from the anodic and cathodic slopes of the linear plots of anodic (*i*_A_) and cathodic current (*i*_C_) versus the potential scan rate (ν), respectively.

## Supporting Information Available

Equations used to calculate the measured parameters; figures for the supporting studies; and tables for the numerical analysis of electrocatalytic data (PDF)
Flat and compact fabric-like CNTR after compression revealing its flexible and lightweight nature (Supporting Video SV1_CNTR) (MP4)
Bifunctional catalytic activity of NiO QD-coated CNTR electrodes, employing as both the anode and the cathode in a 2E configuration, for the OWS performances in a N_2_-saturated alkaline medium (SV2_OWS) (MP4)
