# Supplementary material for: Flexible Bifunctional Electrode for Alkaline Water Splitting with Long-Term Stability
Source: ACS Appl Mater Interfaces. 2024 Mar 1;16(10):12339–52. doi: 10.1021/acsami.3c12944 (PMC10941191; doi:10.1021/acsami.3c12944)
Supplement: Supplementary file 1 — am3c12944_si_001.pdf [file am3c12944_si_001.pdf]

Supporting Information

Flexible Bifunctional Electrode for Alkaline Water Splitting with Long-Term Stability

Abhijit Ganguly,† Ruairi J. McGlynn,† Adam Boies,‡ Paul Maguire,† Davide Mariotti,† and Supriya Chakrabarti*,†

† School of Engineering, Ulster University, Belfast BT15 1AP, Northern Ireland, UK

‡ Department of Engineering, University of Cambridge, Cambridge, CB2 1PZ, UK

* Corresponding author. *E-mail address*: s.chakrabarti@ulster.ac.uk (S. Chakrabarti).

S1. ANALYSIS & EQUATIONS

S1.1. HER Kinetics and Pathways in Acidic and Alkaline Media.

As per classical cathodic HER,[1] the two steps, either Volmer-Tafel (VT) or Volmer-Heyrovsky (VH) pathways, are mainly suggested:

Firstly, the adsorption of intermediate adsorbed hydrogen atom (H_{ads}) on the cathode surface (*) via Volmer reaction (Eqn. S1):

Volmer step:

In acidic medium, $* + H_3O^+ + e^- \rightarrow\ * H_{\mathrm{ads}}$ (S1a)

In alkaline medium, $* + 2H_2O + 2e^- \rightarrow 2 * H_{\mathrm{ads}} + 2OH^-$ (S1b)

Adsorption & discharge step: Tafel slope $= {}^{2.3\mathrm{RT}}/_{\propto \mathrm{F}} \approx 120\ \mathrm{mV/dec}$

In acidic media, the source of protons is H_3O^+ for hydrogen evolution. Firstly, the adsorption of intermediate adsorbed hydrogen atoms (H_{ads}) on the cathode surface via Volmer reaction (Eqn. S1a).

In alkaline media, the source of protons is simply H_2O, the dissociation of which first takes place via Eqn. S1b producing the oxygen species (OH^-) at the electrode/electrolyte interface.

Next, the second step would follow either Heyrovsky desorption (Eqn. S2) via a reaction of H_{ads} intermediates with proton (in acid media, Eqn. S2a) or water molecules (in alkaline, Eqn. S2b) and electron transferred from the electrode surface; or via recombination of the two H_{ads} on the electrode surface (Tafel desorption, Eqn. S3) to generate H_2 gas molecule:

Heyrovsky step:

In acidic medium, $* H_{ads} + H_3O^+ + e^- \rightarrow \mathrm{H_2}\uparrow + *$ (S2a)

In alkaline medium, $H_2O + * H_{ads} + e^- \rightarrow \mathrm{H_2} \uparrow + OH^- + *$ (S2b)

Desorption step: Tafel slope $= {}^{2.3\mathrm{RT}}/_{(1+\propto)\mathrm{F}} \approx 40\ \mathrm{mV/dec}$

Tafel step:

$$2 * H_{ads} \rightarrow \mathrm{H_2}\uparrow + 2 * \quad \text{(S3)}$$

$$\text{Recombination step: Tafel slope} = {}^{2.3\mathrm{RT}}/_{2\mathrm{F}} \approx 30\ \mathrm{mV/dec}$$

Tafel analysis and calculation of Tafel slope (b_C or b_A). For Tafel analysis, the polarisation data (LSV: potential, *E* vs. current density, *j*) would be replotted to create the Tafel plot of overpotential (η) vs. log(*j*), following the Tafel equation:[1]

$$\eta = a + b_C \log(j) \quad \text{(S4)}$$

where b_C stands the cathodic Tafel slope for HER, while the anodic slope, for OER b_A represents the anodic Tafel slope.

The overpotential (η) is estimated by:

For HER, $\eta = E^0 - E$, where E^0 represents $E^0(H_2O/H_2) = 0\ V\ vs.RHE$

For OER, $\eta = E^0 - E$, where E^0 represents $E^0(O_2/H_2O) = 1.23\ V\ vs.RHE$

The exchange current density (j_0), the intercept in the log(*j*) axis estimated by extrapolating the approximated linear potion of the Tafel plot, is a quantitative measure of the rate of reaction at equilibrium (i.e., at $\eta = 0$).

Calculation of electrochemically active surface area (*ECSA*). The estimation of electrochemically active surface area (*ECSA*) was conducted by measurement of the double-layer capacitance in a potential region with no Faradaic response following McCory et al.'s methodology:[2]

$$ECSA = {}^{C_{\mathrm{dl}}}/_{C_{\mathrm{S}}} \quad \text{(S5)}$$

where C_{dl} represents the measured double-layer capacitance of the working electrodes, and C_S is the theoretical specific capacitance of an atomically smooth planar surface of the material per unit area under identical electrolyte conditions. Here, we use C_S = 0.035 mF/cm^2 in H_2SO_4 and C_S = 0.040 mF/cm^2 in KOH based on the typical values reported by McCory et al.[2]

The C_{dl} value was estimated by performing the CV in the non-Faradaic region and averaged from the anodic and cathodic slopes of the linear plots of anodic (i_A) and cathodic current (i_C) versus the potential scan rate (ν), respectively.[2]

$$C_{dl} = \frac{1}{2}\left(\frac{\delta i_A}{\delta \nu} + \frac{\delta i_C}{\delta \nu}\right) \quad \text{(S6)}$$

where ${\delta i_A}/{\delta \nu}$ and ${\delta i_C}/{\delta \nu}$ represent the anodic and cathodic slopes, respectively.

Successively, the roughness factor (*RF*) can be estimated by:[2]

$$RF = {ECSA}/{GSA} \quad \text{(S7)}$$

where *GSA* represent the geometric surface area (≈ 0.16 cm^2) of the laminated electrodes of pristine and NiO QDs coated CNTR (Φ = 4.5 mm). For Pt wire, the *GSA* represents the immersed area of the wire, around 0.395 cm^2.

S1.2. OER Kinetics and Pathways in Alkaline Media.

In alkaline media, the OER happens following a 4e$^-$ pathway initiated with hydroxide (OH^-) anion:[3,4]

$$* + OH^- \rightarrow\ *OH_{\mathrm{ads}} + \mathrm{e}^- \quad \text{(S8)}$$

$$*OH_{ads} + OH^- \rightarrow\ *O_{ads} + H_2O + e^- \quad \text{(S9)}$$

$$*O_{ads} + OH^- \rightarrow *OOH_{ads} + e^- \quad \text{(S10)}$$

$$*OOH_{ads} + OH^- \rightarrow O_2 \uparrow + H_2O + e^- + * \quad \text{(S11)}$$

S2. FIGURES AND TABLES

S2.1. Macroscopically Assembled CNT Ribbons, CNTR.

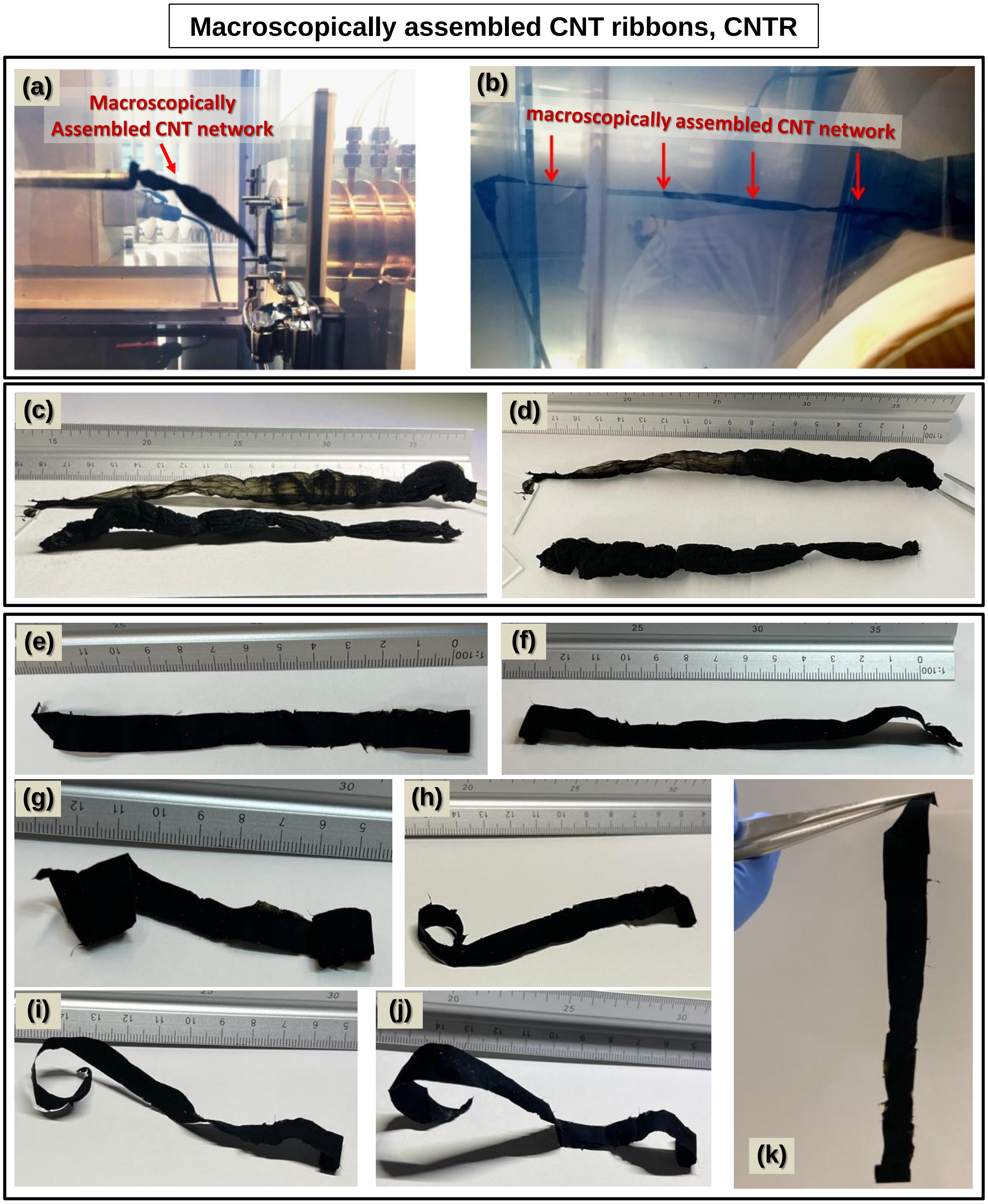

Figure S1. (a-b) Direct dragging of ribbon-like macroscopically assembled CNT network from the furnace chamber. (c-d) Representative digital images of a typical as-synthesised 3D ensemble of

randomly oriented CNT network. (e-k) Representative images of the flat and compact fabric-like CNTR after compression revealing its flexible and light-weight nature.

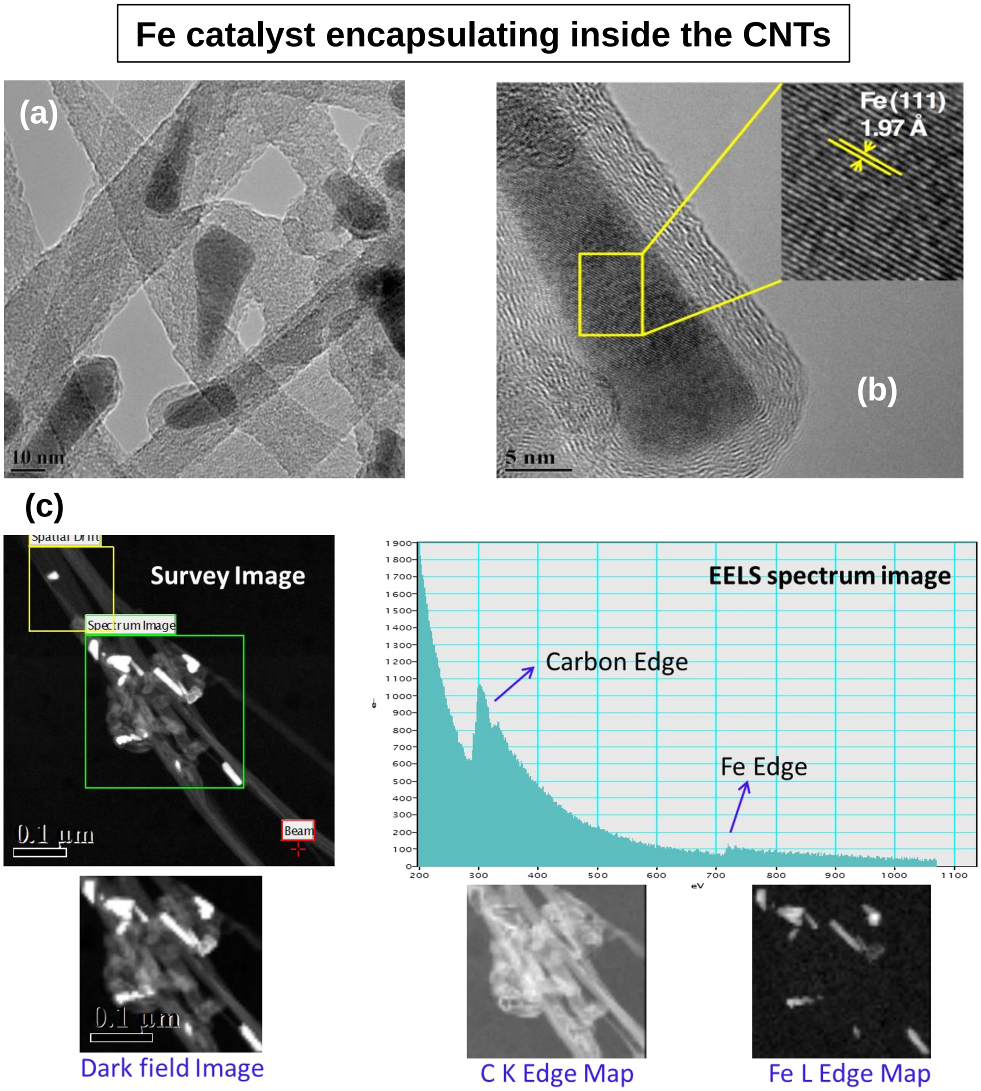

Figure S2. (a) High-resolution TEM image showing the Fe catalyst particles encapsulated in the nanotubes. (b) HRTEM image of an individual CNT showing the encapsulated Fe catalyst; the inset shows the lattice spacing of the Fe catalyst particle. (c) EELS spectrum of the pristine CNTR.

S2.2. Fabrication and Testing of Laminated Electrodes (*CNTR* and *NiO@CNTR*)

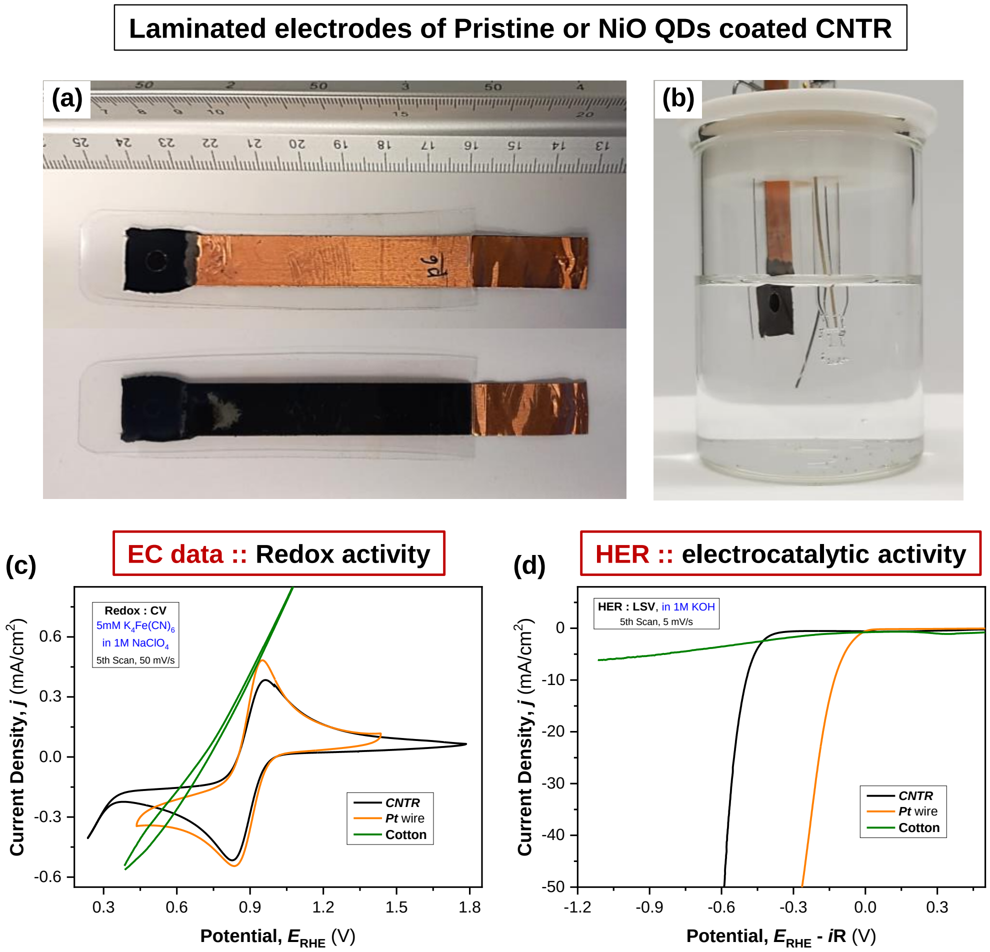

Figure S3. Digital images of (a) a representative laminated electrode of pristine or NiO QDs coated CNTR and (b) the experimental setup used for the electrocatalytic measurements. (c) Typical CV spectra comparing the $Fe(CN)_6^{3-/4-}$ redox activities of pristine ***CNTR*** and ***Pt*** wire electrodes, measured in 1M $NaClO_4$; and (d) Typical electrocatalytic activity of HER in 1M KOH, comparing with a similarly-laminated electrode made of cotton fabric: confirming no wetting of the CNTR sample while immersed in an electrolyte and no leaking of the electrolyte (through the window or edge of the laminated electrode), hence no direct interference of the metal-contact (Cu tape or Ni conducting paste used for the laminated electrode) with the electrolyte solution or the electrocatalytic reaction.

Testing and optimisation of laminated electrodes. Prior to the electrocatalytic measurements (experimental setup, Fig. S3b), the laminated CNTR electrodes were tested and optimised by employing conventional electrochemical characterisations, such as redox (Fig. S3c, using $Fe(CN)_6^{3-/4-}$ based redox in neutral solution) and electrocatalytic (Fig. S3d, HER in alkaline electrolyte) activities. Here, it was confirmed that there was no "wetting" of the CNTR electrode during the electrochemical operations (Figs. S3b and S3d) due to inherent hydrophobic nature of CNTs. For comparison we used laminated electrode made of cotton fabric and performed similar electrochemical measurements (results shown in Figs. S3b and S3d). Here also no "leaking" of the electrolyte through the window or edge of the laminated electrode was verified. If there is any "leaking" or "wetting", there would be an undesirable interference of the metal contact (adhesive Cu tape or Ni conducting paste) with the electrolyte solution during the electrochemical operations. As a result, it can affect the resulting redox or catalytic spectra, producing tilted CV (current varying linearly with potential) and sloppy LSV (without any noticeable drop in the current), similar to those shown by the electrode made of cotton fabric.

S2.3. Nickel Oxide (NiO) Quantum Dots (QDs).

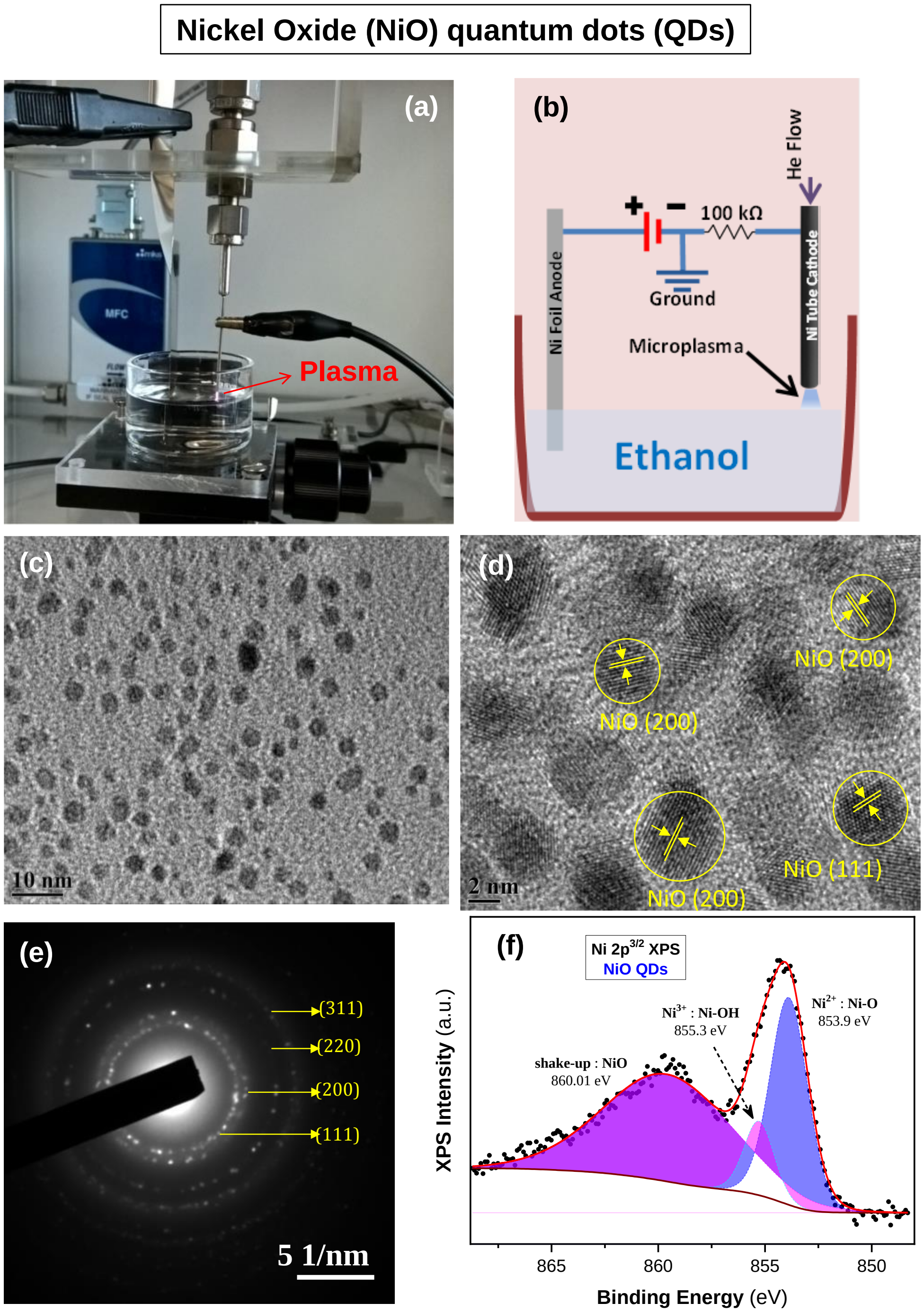

Figure S4. (a) Digital image and (b) schematic diagram of the PiNE experimental setup used for the synthesis of NiO QDs. (c) TEM micrograph and (d) HRTEM image of NiO QDs showing the crystallinity and lattice spacing. (e) SAED pattern of NiO QDs. (f) High-resolution XPS spectrum of Ni $2p^{3/2}$ of NiO QDs synthesized by PiNE.

S2.4. Designation of the Electrode samples.

Table S1. Designation of the proposed electrode samples used in the study.

Electrode ID	Description of Electrodes	Added NiO QDs colloids		(Maximum) Loading of NiO QDs	Electrode Surface (Window) Area
		Concentration	Volume		
CNTR	Pristine CNT ribbon	- -	- -	- -	≈ 0.16 cm^2
NiO*-0.1@*CNTR	***NiO*-0.1** coated CNTR	≈ 6.67 µg/mL	20 µL	8.3 µg/cm^2	≈ 0.16 cm^2
NiO*-0.5@*CNTR	***NiO*-0.5** coated CNTR	≈ 33.33 µg/mL	20 µL	42 µg/cm^2	≈ 0.16 cm^2
NiO*-1.0@*CNTR	***NiO*-1.0** coated CNTR	≈ 66.67 µg/mL	20 µL	83 µg/cm^2	≈ 0.16 cm^2
Pt wire	commercial Pt wire (BASi)	- -	- -	- -	≈ 0.39 cm^2

S2.5. Alkaline-friendly and Bifunctional Nature of pristine *CNTR* and *NiO@CNTR* Electrodes.

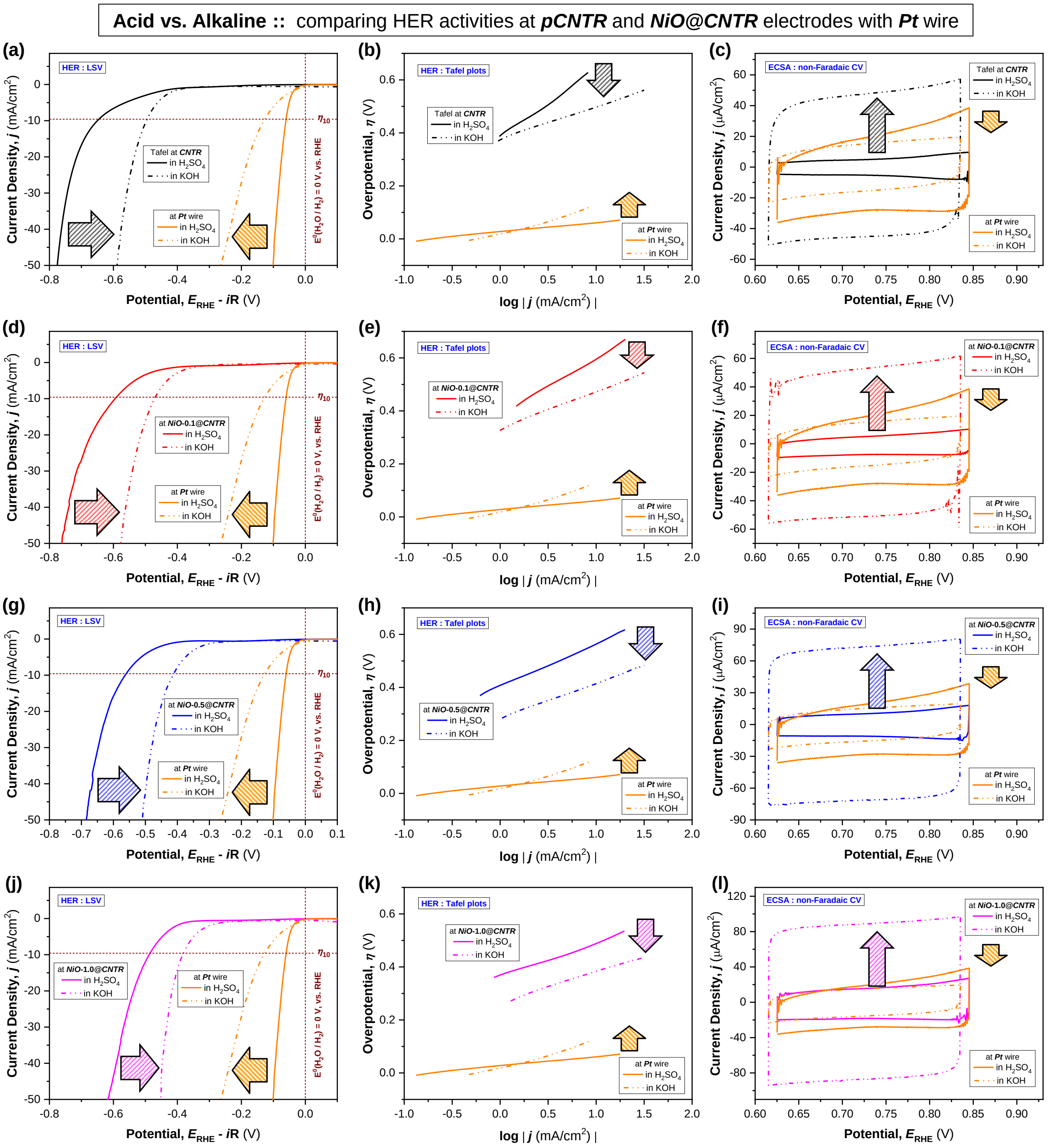

Figure S5. Acid (0.5M H_2SO_4) vs alkaline (1M KOH) electrolytes: Comparison of HER performances of pristine and NiO QDs coated CNTR electrodes with ***Pt*** wire electrode: (a-c) ***CNTR***, (d-f) ***NiO-0.1@CNTR***, (g-i) ***NiO-0.5@CNTR*** and (j-l) ***NiO-1.0@CNTR*** electrodes. Here, the left column represents LSV plots (*iR* corrected, at a potential scan rate (*ν*) of 5 mV/s), and their respective Tafel plots are

demonstrated in the central column, while the right column displays the CV plots performed in the non-Faradaic potential region at $\nu = 50$ mV/s. Respective data measured at commercial ***Pt*** wire electrode are presented for comparison.

S2.6. CV Study in non-Faradaic Potential Region.

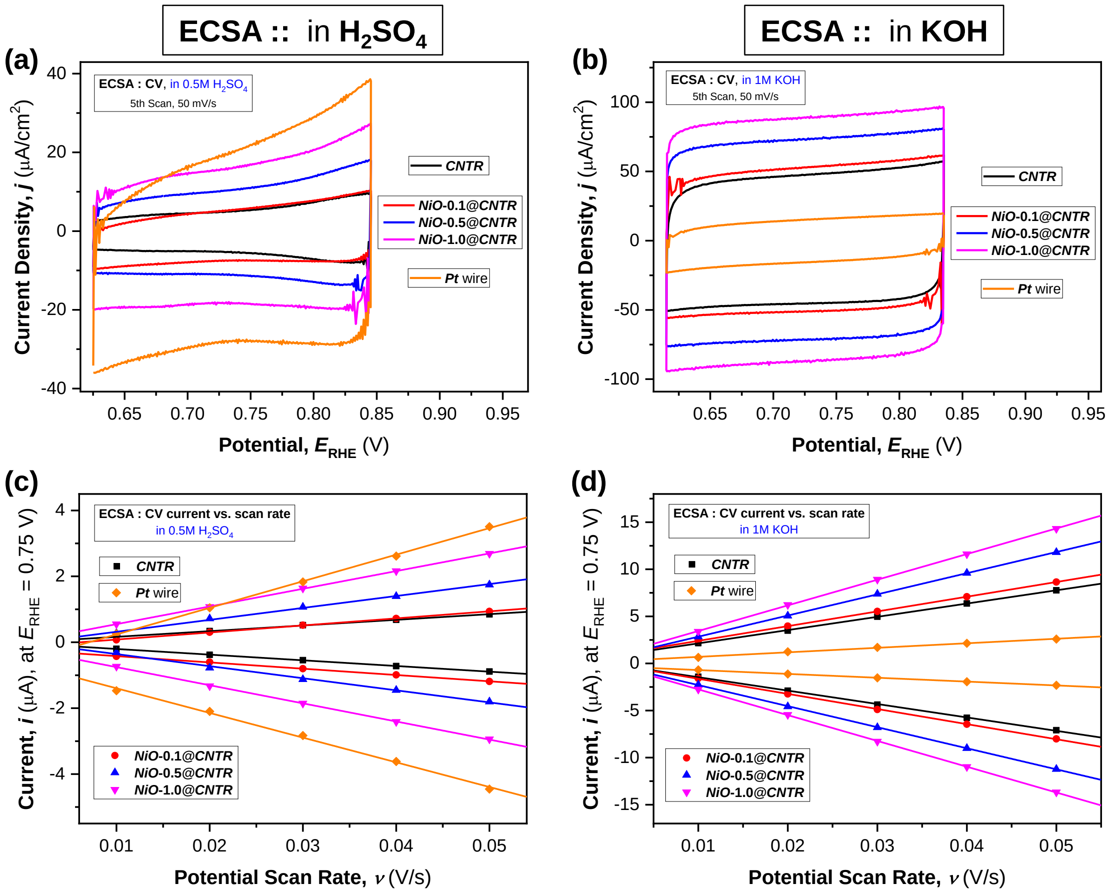

Figure S6. Comparison of cyclic voltammograms of pristine (***CNTR***) and NiO QDs coated (***NiO@CNTR***) electrodes, performed in the non-Faradaic region (potential scan rate, $\nu = 50$ mV/s), an aqueous (a) acidic (0.5M H_2SO_4) and (b) alkaline (1M KOH) media. Corresponding plots of cathodic (i_C) and anodic current (i_A) as the function of potential scan rate (υ), measured in (c) 0.5M H_2SO_4 and (d) 1M KOH, respectively. Respective data measured at commercial ***Pt*** wire electrode are presented for comparison.

S2.7. Stability of alkaline OWS performance: data reproducibility

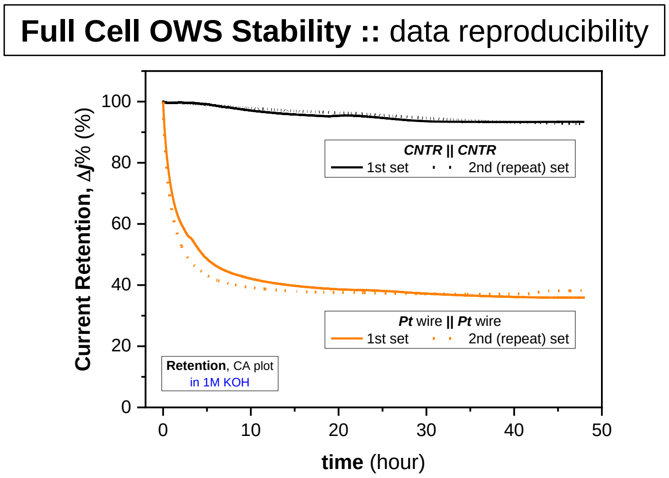

Figure S7. Long-term stability of alkaline OWS (chronoamperometry, CA), of pristine ***CNTR*** || ***CNTR*** and ***Pt*** wire || ***Pt*** wire electrolysers, monitoring the current retention at the respective E_{10} (recorded at a current density of 10 mA/cm^2 from the initial OWS plots (before CA, Fig. 6b)). Performances of both electrolysers were repeated (using a fresh set of electrodes) to evaluate the reproducibility of results.

S2.8. Electrocatalytic characteristics of *CNTR* and *NiO@CNTR* electrodes.

Table S2. Electrocatalytic characteristics for H_2 evolution reaction (**HER**) in **0.5M H_2SO_4**.

Electrode ID	η_{10} (mV)	b_C (mV/decade)	j_0 (mA/cm^2)	C_{dl} (mF)	*ECSA* (cm^2)	RF
CNTR	649.39 ± 5	210.99 ± 1.36	13.277	0.017	0.492	3.092
NiO*-0.1@*CNTR	594.38 ± 5	195.96 ± 2.13	9.465	0.02	0.579	3.639
NiO*-0.5@*CNTR	563.2 ± 5	144.61 ± 0.47	1.503	0.036	1.041	6.543
NiO*-1.0@*CNTR	488.11 ± 5	104.43 ± 0.64	0.274	0.054	1.552	9.759
Pt wire	60.81 ± 5	34.24 ± 1.38	153.89	0.078	2.222	5.631

Table S3. Electrocatalytic characteristics for H_2 evolution reaction (**HER**) in **1M KOH**.

Electrode ID	η_{10} (mV)	b_C (mV/decade)	j_0 (mA/cm^2)	C_{dl} (mF)	*ECSA* (cm^2)	RF
CNTR	496.51 ± 5	113.21 ± 0.59	0.414	0.142	3.541	22.263
NiO*-0.1@*CNTR	471.47 ± 5	104.78 ± 2.24	3.083	0.158	3.96	24.9
NiO*-0.5@*CNTR	414.05 ± 5	103.16 ± 1.12	4.502	0.224	5.606	35.25
NiO*-1.0@*CNTR	383.24 ± 5	94.79 ± 2.26	4.512	0.273	6.828	42.93
Pt wire	129.08 ± 5	87.93 ± 4.7	637.7	0.045	1.112	2.817

Table S4. Electrocatalytic characteristics for O_2 evolution reaction (**OER**) in **1M KOH**.

Electrode ID	η_{10} (mV)	b_A (mV/decade)	j_0 (mA/cm^2)	C_{dl} (mF)	*ECSA* (cm^2)	RF
CNTR	405 ± 5	39.23 ± 1.04	0.00049	0.142	3.541	22.263
NiO*-0.1@*CNTR	341 ± 5	44.1 ± 0.35	0.00038	0.158	3.96	24.9
NiO*-0.5@*CNTR	315 ± 5	39.14 ± 0.51	0.00013	0.224	5.606	35.25
NiO*-1.0@*CNTR	256 ± 5	33.52 ± 0.34	0.00024	0.273	6.828	42.93
Pt wire	829 ± 5	119.39 ± 1.14	0.00135	0.045	1.112	2.817

η_{10}**:** Overpotential values @ 10 mA/cm^2, respectively, measured from LSV plots
b_A and b_C**:** Anodic and cathodic Tafel slope, respectively; and j_0**:** exchange current density, measured from Tafel plots
C_{dl}**:** Double-layer capacitance; ***ECSA*:** electrochemical active area; and ***RF*:** roughness factor, estimated from CV measurements, performed in non-Faradaic potential region

S2.9. Literature Comparison of Overall Water Splitting (OWS) Performance

Table S5. Brief literature comparison of **OWS** performance of various **bifunctional electrocatalysts**, reported in 1 M alkaline electrolytes (KOH or NaOH).

Catalyst		Electrode support	Catalyst Loading (mg/cm^2)	OWS performance		Long-term stability test			Ref
**Anode**	**Cathode**			E_{10} (V)	**Scan rate** (mV/s)	**Duration** (hour)	Set ***E*** or ***j***	**Retention**	
NiO$_{1.0}$@CNTR	***NiO$_{1.0}$@CNTR***	**CNTR**	0.083	1.80		**100 h**	1.80 V	**~99.92%**	**Our work**
				1.81		48 h	1.81 V	**~100.2%**	
NiO$_{1.0}$@CNTR	***Pt*** wire		0.083	1.63	5 mV/s	48 h	1.63 V	~57.1%	
CNTR	***CNTR***	– –	– –	2.11		48 h	2.11 V	~93.4%	
Pt wire	***Pt*** wire	– –	– –	2.15		48 h	2.15 V	~35.9%	
**NiO NSs**	**α-NiO NSs**	**NF**	0.5266	E_3 >1.8	5 mV/s	– –	– –	– –	5
**α-NiOOH NSs**	**α-NiOOH NSs**	**NF**	0.7351	E_4 >1.75	5 mV/s	– –	– –	– –	
**NiO/GC**	**NiO/GC**	**NF**	~2.0	1.7	– –	10 h	2 V	*~100%	6
**NiO_x-AC**	**NiO_x-AC**	**CP**	~2.0	1.688	5 mV/s	5 h	10 mA/cm^2	*~97.5%	7
**NF**	**NF**	– –	– –	*1.92	1 mV/s	– –	– –	– –	8
**$Ni(OH)_2$ NSs**	**$Ni(OH)_2$ NSs**	**NF**	– –	*1.82	1 mV/s	– –	– –	– –	
**Co_3O_4 NCs**	**Co_3O_4 NCs**	**CFP**	0.35	1.91	5 mV/s	– –	– –	– –	9
**Co_3O_4**	**Co_3O_4**	**Ti** mesh	– –	1.872	5 mV/s	– –	– –	– –	10
**VO-Co_3O_4**	**VO-Co_3O_4**	**Ti** mesh	– –	1.834	5 mV/s	– –	– –	– –	
**N/S-V_O-Co_3O_4**	**N/S-V_O-Co_3O_4**	**Ti** mesh	– –	1.715	5 mV/s	60 h	10 mA/cm^2	*~94%	
**$Ni_{0.5}Se$ NCs**	**$Ni_{0.75}Se$ NCs**	**CP**	0.6	1.73	5 mV/s	10 h	10 mA/cm^2	*≈87.1%	11
**W-$Ni_{12}P_5$**	**W-$Ni_{12}P_5$**	**CC**	– –	1.73	5 mV/s	22h	2 V	*~100%	12
**Ni_5P_4 film**	**Ni_5P_4 film**	**Ni foil**	*3.47	1.70 $	10 mV/s	– –	– –	– –	13
**NF**	**NF**	– –	– –	1.93	2 mV/s	– –	– –	– –	14
**NiSe NWs**	**NiSe NWs**	**NF**	2.8	1.63	2 mV/s	10 h	20 mA/cm^2	*100.5%	
**CoP/rGO**	**CoP/rGO**	**CFP**	~0.28	1.70	5 mV/s	– –	– –	– –	15
**CoP NCs**	**CoP NCs**	**CC**	2.0	1.75	2 mV/s	– –	– –	– –	16
**CoP NFs**	**CoP NFs**	**CC**	2.0	1.65	2 mV/s	30 h	1.7 V	*~96%	
**CoP NPs**	**CoP NPs**	**NF**	~1.2	1.75	2 mV/s	– –	– –	– –	17

**S:CoP NPs**	**S:CoP NPs**	**NF**	~1.2	1.62	2 mV/s	20 h	1.8 V	~100%	
**NiO/Ni-CNT**	**NiFe LDH**	**NF**	8	*1.41	1 mV/s	24 h	20 mA/cm^2	*~100%	[18]
**Ni/Ni_8P_3**	**Ni/Ni_8P_3**	**NF**	10.58	1.61	5 mV/s	4 h	10 mA/cm^2	*~99.1%	[19]
**3D Ni_2P/Ni**	**3D Ni_2P/Ni**	**NF**	– –	1.49	2 mV/s	20 h	20 mA/cm^2	*~100%	[20]
**Ni/$Ni(OH)_2$/CW**	**Ni/$Ni(OH)_2$/CW**	**– –**	– –	1.74	5 mV/s	2000 CV	10 mA/cm^2	*~97.7%	[21]
**NiFe LDH**	**NiFe LDH**	**NF**	– –	1.7	1 mV/s	10 h	1.8 V	*76%	[8]
**$NiCo_2O_4$ NWs**	**$NiCo_2O_4$ NWs**	**CC**	– –	1.98	2 mV/s	– –	– –	– –	[22]
**$NiCo_2S_4$ NA**	**$NiCo_2S_4$ NA**	**CC**	0.43	1.68	5 mV/s	10 h	14 mA/cm^2	*~100%	
**$NiFeO_x$ NPs**	**$NiFeO_x$ NPs**	**CFP**	~1.6	*1.88	5 mV/s	100 h	10 mA/cm^2	*~100%	[23]
**Li^+-$NiFeO_x$ NPs**	**Li^+-$NiFeO_x$ NPs**	**CFP**	~1.6	*1.61	5 mV/s	100 h		*~100%	
**Li^+-$NiFeO_x$ NPs**	**Li^+-$NiFeO_x$ NPs**	**CFP**	3.0	1.51	5 mV/s	200 h		*~100%	
**NiCoP/rGO**	**NiCoP/rGO**	**CFP**	0.15	1.59	5 mV/s	75 h	1.59 V	*~100%	[24]
**CC**	**CC**	**– –**	– –	1.83	5 mV/s	– –	– –	– –	[25]
**CoN/Ni/NiO NPs**	**CoN/Ni/NiO NPs**	**CC**	– –	1.56	– –	12 h	10 mA/cm^2	*~75%	
**$Fe_{0.25}Co_{0.75}$/CC**	**$Fe_{0.25}Co_{0.75}$/CC**	**– –**	– –	1.66	1 mV/s	100 h	10 mA/cm^2	*~97.6%	[26]
**FeCo/Co_2P/Fe_2P @NPC**	**FeCo/Co_2P/Fe_2P @NPC**	**CC**	~1.0	1.55	5 mV/s	24 h	1.58 V	*~75%	[27]

***E_{10}* :** Cell (**OWS**) potential at 10 mA/cm^2

*** :** *E*stimated from the published work | **$:** using rotating disk electrode @ 2000 rpm

NSs: Nanosheets | **NCs:** Nanocrystals | **NPs:** Nanoparticles

NWs: Nanowires | **NA:** Nanowires array | **LDH:** layered double hydroxides

NF: Ni foam | **CP:** Carbon paper | **CFP:** Carbon Fiber paper | **CC:** Carbon cloth

NiO/GC: NiO NPs infused graphitic carbon (GC) nanosheet | **NiO_x-AC:** Ni/NiO composite with N-doped activated carbon

α-NiOOH: vertically aligned orthorhombic α-NiOOH nanosheet arrays | **W-$Ni_{12}P_5$:** 15% W-doped $Ni_{12}P_5$ Nanoparticles

N/S-VO-Co_3O_4: In situ filling of the O vacancies (VO) with dual N and S heteroatoms in Co_3O_4 electrodeposited on Ti mesh

rGO : Reduced Graphene oxides | **S:CoP:** Sulfur-doped CoP | **Li^+-NiFeOx:** Li+-intercalated ultra-small NiFeOx

3D Ni_2P/Ni: hierarchically porous urchin-like Ni_2P microsphere superstructures

Ni/$Ni(OH)_2$/CW: nanosheet-shaped Ni/α&β-$Ni(OH)_2$ arrays electrodeposited on a carbonized wood (CW) electrode

FeCo/Co_2P/Fe_2P@NPC: FeCo-rich phosphides nanoparticles and porous N,P co-doped carbon network

References

(1) Bard, A. J.; Faulkner, L. R. *Electrochemical Methods: Fundamentals and Applications*, 2nd ed.; John Wiley and Sons, **2001**.

(2) McCrory, C. C. L.; Jung, S.; Peters, J. C.; Jaramillo, T. F. Benchmarking Heterogeneous Electrocatalysts for the Oxygen Evolution Reaction. *J. Am. Chem. Soc.* **2013**, *135* (45), 16977–16987. https://doi.org/10.1021/ja407115p.

(3) Shinagawa, T.; Garcia-Esparza, A. T.; Takanabe, K. Insight on Tafel Slopes from a Microkinetic Analysis of Aqueous Electrocatalysis for Energy Conversion. *Sci. Rep.* **2015**, *5* (1), No. 13801. https://doi.org/10.1038/srep13801.

(4) Gu, Y.; Chen, S.; Ren, J.; Jia, Y. A.; Chen, C.; Komarneni, S.; Yang, D.; Yao, X. Electronic Structure Tuning in Ni_3FeN/r-GO Aerogel toward Bifunctional Electrocatalyst for Overall Water Splitting. *ACS Nano* **2018**, *12* (1), 245–253. https://doi.org/10.1021/acsnano.7b05971.

(5) Zhang, Q.; Zhang, C.; Liang, J.; Yin, P.; Tian, Y. Orthorhombic α-NiOOH Nanosheet Arrays: Phase Conversion and Efficient Bifunctional Electrocatalysts for Full Water Splitting. *ACS Sustain. Chem. Eng.* **2017**, *5* (5), 3808–3818. https://doi.org/10.1021/acssuschemeng.6b02788.

(6) Veeramani, K.; Shanmugapriya, S.; Young Kim, J.; Surendran, S.; Jun Moon, D.; Janani, G.; Cyril Jesudass, S.; Mahadik, S.; Choi, H.; Jung, P.; Goo Kim, I.; Heo, J.; Hong, K.; Kim, T.-H.; Il Park, Y.; Sim, U. Synergistically Enhanced Electrocatalytic Performance of NiO Infused Crystalline Graphitic Carbon towards Overall Water Splitting. *Mater. Lett.* **2024**, *356*, No. 135593. https://doi.org/https://doi.org/10.1016/j.matlet.2023.135593.

(7) Hoang, V. C.; Dinh, K. N.; Gomes, V. G. Hybrid Ni/NiO Composite with N-Doped Activated Carbon from Waste Cauliflower Leaves: A Sustainable Bifunctional Electrocatalyst for Efficient Water Splitting. *Carbon N. Y.* **2020**, *157*, 515–524. https://doi.org/https://doi.org/10.1016/j.carbon.2019.09.080.

(8) Luo, J.; Im, J.-H.; Mayer, M. T.; Schreier, M.; Nazeeruddin, M. K.; Park, N.-G.; Tilley, S. D.; Fan, H. J.; Grätzel, M. Water Photolysis at 12.3% Efficiency via Perovskite Photovoltaics and Earth-Abundant Catalysts. *Science.* **2014**, *345* (6204), 1593–1596.

https://doi.org/10.1126/science.1258307.

(9) Du, S.; Ren, Z.; Zhang, J.; Wu, J.; Xi, W.; Zhu, J.; Fu, H. Co_3O_4 Nanocrystal Ink Printed on Carbon Fiber Paper as a Large-Area Electrode for Electrochemical Water Splitting. *Chem. Commun.* **2015**, *51* (38), 8066–8069. https://doi.org/10.1039/C5CC01080B.

(10) Duan, W.; Han, S.; Fang, Z.; Xiao, Z.; Lin, S. In Situ Filling of the Oxygen Vacancies with Dual Heteroatoms in Co_3O_4 for Efficient Overall Water Splitting. *Molecules*. **2023**, *28*, 4134. https://doi.org/10.3390/molecules28104134.

(11) Zheng, X.; Han, X.; Liu, H.; Chen, J.; Fu, D.; Wang, J.; Zhong, C.; Deng, Y.; Hu, W. Controllable Synthesis of Ni_xSe ($0.5 \leq x \leq 1$) Nanocrystals for Efficient Rechargeable Zinc–Air Batteries and Water Splitting. *ACS Appl. Mater. Interfaces* **2018**, *10* (16), 13675–13684. https://doi.org/10.1021/acsami.8b01651.

(12) Ghosh, S.; Kadam, S. R.; Kolatkar, S.; Neyman, A.; Singh, C.; Enyashin, A. N.; Bar-Ziv, R.; Bar-Sadan, M. W Doping in $Ni_{12}P_5$ as a Platform to Enhance Overall Electrochemical Water Splitting. *ACS Appl. Mater. Interfaces* **2022**, *14* (1), 581–589. https://doi.org/10.1021/acsami.1c16755.

(13) Ledendecker, M.; Krick Calderón, S.; Papp, C.; Steinrück, H.-P.; Antonietti, M.; Shalom, M. The Synthesis of Nanostructured Ni_5P4 Films and Their Use as a Non-Noble Bifunctional Electrocatalyst for Full Water Splitting. *Angew. Chemie Int. Ed.* **2015**, *54* (42), 12361–12365. https://doi.org/https://doi.org/10.1002/anie.201502438.

(14) Tang, C.; Cheng, N.; Pu, Z.; Xing, W.; Sun, X. NiSe Nanowire Film Supported on Nickel Foam: An Efficient and Stable 3D Bifunctional Electrode for Full Water Splitting. *Angew. Chemie Int. Ed.* **2015**, *54* (32), 9351–9355. https://doi.org/https://doi.org/10.1002/anie.201503407.

(15) Jiao, L.; Zhou, Y.-X.; Jiang, H.-L. Metal–Organic Framework-Based CoP/Reduced Graphene Oxide: High-Performance Bifunctional Electrocatalyst for Overall Water Splitting. *Chem. Sci.* **2016**, *7* (3), 1690–1695. https://doi.org/10.1039/C5SC04425A.

(16) Ji, L.; Wang, J.; Teng, X.; Meyer, T. J.; Chen, Z. CoP Nanoframes as Bifunctional Electrocatalysts for Efficient Overall Water Splitting. *ACS Catal.* **2020**, *10* (1), 412–419. https://doi.org/10.1021/acscatal.9b03623.

(17) Anjum, M. A. R.; Okyay, M. S.; Kim, M.; Lee, M. H.; Park, N.; Lee, J. S. Bifunctional Sulfur-Doped Cobalt Phosphide Electrocatalyst Outperforms All-Noble-Metal Electrocatalysts in Alkaline Electrolyzer for Overall Water Splitting. *Nano Energy* **2018**, *53*, 286–295. https://doi.org/https://doi.org/10.1016/j.nanoen.2018.08.064.

(18) Gong, M.; Zhou, W.; Tsai, M.-C.; Zhou, J.; Guan, M.; Lin, M.-C.; Zhang, B.; Hu, Y.; Wang, D.-Y.; Yang, J.; Pennycook, S. J.; Hwang, B.-J.; Dai, H. Nanoscale Nickel Oxide/Nickel Heterostructures for Active Hydrogen Evolution Electrocatalysis. *Nat. Commun.* **2014**, *5* (1), No. 4695. https://doi.org/10.1038/ncomms5695.

(19) Chen, G.-F.; Ma, T. Y.; Liu, Z.-Q.; Li, N.; Su, Y.-Z.; Davey, K.; Qiao, S.-Z. Efficient and Stable Bifunctional Electrocatalysts Ni/Ni_xM_y (M = P, S) for Overall Water Splitting. *Adv. Funct. Mater.* **2016**, *26* (19), 3314–3323. https://doi.org/https://doi.org/10.1002/adfm.201505626.

(20) You, B.; Jiang, N.; Sheng, M.; Bhushan, M. W.; Sun, Y. Hierarchically Porous Urchin-Like Ni_2P Superstructures Supported on Nickel Foam as Efficient Bifunctional Electrocatalysts for Overall Water Splitting. *ACS Catal.* **2016**, *6* (2), 714–721. https://doi.org/10.1021/acscatal.5b02193.

(21) Yang, J. Y.; Ahn, J. G.; Ko, B.; Park, T.; Hong, S.-J.; Han, D. K.; Lee, D.; Li, C. A.; Song, S. H. Green and Sustainable Bifunctional Carbonized Wood Electrodes Decorated with Controlled Nickel/α(β)-Nickel(II) Hydroxide to Boost Overall Water Splitting. *J. Mater. Chem. A* **2023**, *11* (48), 26672–26680. https://doi.org/10.1039/D3TA05519A.

(22) Liu, D.; Lu, Q.; Luo, Y.; Sun, X.; Asiri, A. M. $NiCo_2S_4$ Nanowires Array as an Efficient Bifunctional Electrocatalyst for Full Water Splitting with Superior Activity. *Nanoscale* **2015**, *7* (37), 15122–15126. https://doi.org/10.1039/C5NR04064G.

(23) Wang, H.; Lee, H.-W.; Deng, Y.; Lu, Z.; Hsu, P.-C.; Liu, Y.; Lin, D.; Cui, Y. Bifunctional Non-Noble Metal Oxide Nanoparticle Electrocatalysts through Lithium-Induced Conversion for Overall Water Splitting. *Nat. Commun.* **2015**, *6* (1), No. 7261. https://doi.org/10.1038/ncomms8261.

(24) Li, J.; Yan, M.; Zhou, X.; Huang, Z.-Q.; Xia, Z.; Chang, C.-R.; Ma, Y.; Qu, Y. Mechanistic Insights on Ternary $Ni_{2-x}Co_xP$ for Hydrogen Evolution and Their Hybrids with Graphene

as Highly Efficient and Robust Catalysts for Overall Water Splitting. *Adv. Funct. Mater.* **2016**, *26* (37), 6785–6796. https://doi.org/https://doi.org/10.1002/adfm.201601420.

(25) Wang, D.; Zhang, C.; Hu, J.; Zhuang, T.; Lv, Z. Nitriding-Reduction Fabrication of Coralloid CoN/Ni/NiO for Efficient Electrocatalytic Overall Water Splitting. *J. Colloid Interface Sci.* **2024**, *655*, 217–225. https://doi.org/https://doi.org/10.1016/j.jcis.2023.11.018.

(26) Walendzik, I.; Kordek-Khalil, K.; Rutkowski, P. Quick-to-Synthesize Hybrid Electrodes Composed of Activated Carbon Cloth with Co/Fe-Based Films as Bifunctional Electrocatalysts for Water Splitting. *Int. J. Hydrogen Energy* **2023**, *48* (66), 25741–25754. https://doi.org/https://doi.org/10.1016/j.ijhydene.2023.03.092.

(27) Wang, B.; Liu, Q.; Yuan, A.; Shi, Q.; Jiang, L.; Yang, W.; Yang, T.; Hou, X. A Facile and Green Strategy for Mass Production of Dispersive FeCo-Rich Phosphides@N,P-Doped Carbon Electrocatalysts toward Efficient and Stable Rechargeable Zn-Air Battery and Water Splitting. *J. Mater. Sci. Technol.* **2024**, *182*, 1–11. https://doi.org/https://doi.org/10.1016/j.jmst.2023.08.073.
